# Recurrent Rearrangement during Adaptive Evolution in an Interspecific Yeast Hybrid Suggests a Model for Rapid Introgression

**DOI:** 10.1371/journal.pgen.1003366

**Published:** 2013-03-21

**Authors:** Barbara Dunn, Terry Paulish, Alison Stanbery, Jeff Piotrowski, Gregory Koniges, Evgueny Kroll, Edward J. Louis, Gianni Liti, Gavin Sherlock, Frank Rosenzweig

**Affiliations:** 1 Department of Genetics, Stanford University School of Medicine, Stanford, California, United States of America; 2 Division of Biological Sciences, University of Montana, Missoula, Montana, United States of America; 3 Chemical Genomics Research Group, RIKEN Advance Science Institute, Wako, Japan; 4 Center of Genetics and Genomics, Queen's Medical Centre, University of Nottingham, Nottingham, United Kingdom; Washington University School of Medicine, United States of America

## Abstract

Genome rearrangements are associated with eukaryotic evolutionary processes ranging from tumorigenesis to speciation. Rearrangements are especially common following interspecific hybridization, and some of these could be expected to have strong selective value. To test this expectation we created *de novo* interspecific yeast hybrids between two diverged but largely syntenic *Saccharomyces* species, *S. cerevisiae* and *S. uvarum*, then experimentally evolved them under continuous ammonium limitation. We discovered that a characteristic interspecific genome rearrangement arose multiple times in independently evolved populations. We uncovered nine different breakpoints, all occurring in a narrow ∼1-kb region of chromosome 14, and all producing an “interspecific fusion junction” within the *MEP2* gene coding sequence, such that the 5′ portion derives from *S. cerevisiae* and the 3′ portion derives from *S. uvarum*. In most cases the rearrangements altered both chromosomes, resulting in what can be considered to be an introgression of a several-kb region of *S. uvarum* into an otherwise intact *S. cerevisiae* chromosome 14, while the homeologous *S. uvarum* chromosome 14 experienced an interspecific reciprocal translocation at the same breakpoint within *MEP2*, yielding a chimaeric chromosome; these events result in the presence in the cell of two *MEP2* fusion genes having identical breakpoints. Given that *MEP2* encodes for a high-affinity ammonium permease, that *MEP2* fusion genes arise repeatedly under ammonium-limitation, and that three independent evolved isolates carrying *MEP2* fusion genes are each more fit than their common ancestor, the novel *MEP2* fusion genes are very likely adaptive under ammonium limitation. Our results suggest that, when homoploid hybrids form, the admixture of two genomes enables swift and otherwise unavailable evolutionary innovations. Furthermore, the architecture of the *MEP2* rearrangement suggests a model for rapid introgression, a phenomenon seen in numerous eukaryotic phyla, that does not require repeated backcrossing to one of the parental species.

## Author Summary

Interspecific hybridization occurs when two different species mate and produce viable offspring. While hybrid offspring are usually sterile, like the mule, which results from a horse–donkey mating, sometimes they are fertile, creating new species. Indeed, many plant and animal species have arisen via this mechanism. Because interspecific hybridization occurs between different yeast species, and because they are such tractable models, yeast are ideally suited for experimentally investigating the genomic consequences of interspecific hybridization. We created an interspecific yeast hybrid by crossing *S. cerevisiae* and *S. uvarum*, and then studied genomic changes that occurred as it adaptively evolved in a stressful nitrogen-limiting environment. We discovered that a characteristic rearrangement between the parental species' chromosomes evolved independently many times, and always within a particular gene encoding a protein that imports nitrogen into the cell. Evolved hybrids carrying this rearrangement grew faster under nitrogen-limitation than ancestral hybrids, suggesting that the rearrangement is beneficial in nitrogen-poor environments. Our results suggest that having the genomes of two different species within a cell provides novel sources of variation for evolution to act upon, leading to adaptations that could not occur in either parental species.

## Introduction

Eukaryotic genome content and architecture can vary dramatically as populations of organisms evolve, or as populations of cells evolve during disease processes like cancer [1], [2]. Chromosome number may change, resulting in polyploidy and/or aneuploidy, or chromosomes may be restructured by translocations, inversions, deletions and amplifications. A striking example of genomic change, homoploid hybrid speciation, occurs when gametes of closely related species fuse to form viable hybrids. If both parental species have the same number of chromosomes, the homoploid hybrid will contain a “diploid” genome that has the same chromosome number as its ancestors; such hybrids can also be called “F1 hybrids,” as they arise in the first filial generation following hybridization. By contrast, allopolyploid hybrid speciation typically results in a doubling (or more) of the ancestral chromosome number. Although homoploid hybrid speciation has been most commonly observed in plants [3], it has been documented in every eukaryotic Kingdom (e.g., [4]–[7]). In the wild, as well as in brewing and wine-making, both homoploid and allopolyploid hybrid yeast have been isolated whose genomes are wholly or partly derived from two or more different members of the *Saccharomyces* “*sensu stricto*” group [8]–[10]. These *Saccharomyces* species can also be mated in the lab to create *de novo* interspecific hybrids [11]–[13].

In addition to homoploid hybrids that bear one copy of each of their parental species' chromosomes, “introgressive hybridization,” also known as introgression, has been observed among the *sensu stricto* group of *Saccharomyces*. This term was first coined by Anderson and Hubricht in 1938 [14] to denote the infiltration of the “germplasm” of one species into that of another following hybridization and repeated backcrossing. If this region is not selected against, in time it can become established as an “island” of the minor species' genome encompassed within the major species' genome. Introgressive hybridization is thought to be a long-term process, requiring an initial interspecific hybridization event, followed by the repeated backcrossing with only one of its parent species [15]. Since its first description, introgressive hybridization has been identified in numerous eukaryotic phyla (see [16], [17] for reviews). Introgression events have been documented among many of the *Saccharomyces sensu stricto* species, in yeasts isolated from natural environments [11], [18], clinical and animal sources [19], [20], and from wine, beer, and other industrial environments ([8], [21]–[28]; also see [29] for review). Like homoploid and polyploid hybridization, introgression is also considered to be important as a mechanism leading to speciation [30], [31]. Indeed, hybridization and introgression have been suggested as sources of unexpected, extreme ‘transgressive’ phenotypic traits upon which natural selection can act [32], facilitating rapid within-lineage evolution [30].

Although the evolutionary implications of interspecific hybridization in general, and introgressive hybridization in particular, have been appreciated for some time, their molecular bases and relative importance as evolutionary mechanisms among various Kingdoms are incompletely understood [33]. Moreover, while genomic technologies have greatly expanded our understanding of genome content and stability during adaptive evolution, we have limited knowledge of how genomes stabilize following the initial ‘shock’ of interspecific hybridization [34]. Significantly, the actual process of introgressive hybridization has never been captured in action. Budding yeasts of the genus *Saccharomyces* provide an ideal eukaryotic system in which to close these knowledge gaps. Not only do *Saccharomyces* yeasts readily form interspecific hybrids, they also have a relatively simple life cycle, reproduce quickly, tolerate aneuploidy [35] and can be propagated as stable haploids or diploids. Environmental variables and the size and structure of yeast populations can also be controlled experimentally, and because yeasts can be preserved cryogenically, it is possible to compare evolved to ancestral strains or to replicate any stage of an experiment [36].

*Saccharomyces cerevisiae* and *S. uvarum* (previously called *S. bayanus*) are distantly related members of the *Saccharomyces sensu stricto* group, having diverged ∼20 million years ago [37]. Despite having only 80% sequence identity in coding regions and 62% in intergenic regions, the *S. cerevisiae* and *S. uvarum* genomes are largely syntenic, with the exception of 3 large reciprocal translocations and within some regions of their telomeres, where rapid structural evolution has occurred [37], [38]. Because of their synteny and their sequence divergence, which allows their genomes to be distinguished, we experimentally investigated the evolution of an F1 homoploid interspecific hybrid formed between *S. cerevisiae* and *S. uvarum*. We evolved three independent replicate populations of this hybrid under continuous nitrogen limitation, a selective pressure often encountered in wine-making as well as in other ecological settings where *S. uvarum* and *S. cerevisiae* likely occur [39]–[41]. We determined each parental species' contribution to the evolving genomes by array Comparative Genomic Hybridization (aCGH) as well as by whole genome sequencing of select ancestral and evolved hybrid clones. We discovered a recurrent genomic rearrangement in all three independently evolved hybrid populations. This rearrangement ultimately produces two copies of an interspecific *MEP2* fusion gene, which in both *S. cerevisiae* and *S. uvarum* encodes for a high-affinity ammonium permease. In all cases the fusion gene is structured such that the 5′ end of the gene is derived from *S. cerevisiae* sequences and the 3′ end is derived from *S. uvarum* sequences, an evolutionary innovation that could only arise in a hybrid genome. Repeated evolution of this novel fusion gene in independent populations suggests that it is adaptive under nitrogen-poor environments where ammonium is the sole nitrogen source. The architecture of the rearrangement suggests a model for rapid introgression without the need for repeated backcrossing to one of the parental species.

## Results

We created an *S. cerevisiae-S. uvarum* interspecific F1 homoploid hybrid, strain GSY86 (Table 1), by mass-mating a haploid *S. cerevisiae* strain (S288c background) to haploid spores of *S. uvarum* (CBS7001 strain background), as described in Materials and Methods and shown schematically in Figure S1. Experimental populations were founded by GSY86 in three independent vessels, which hereafter we call vessel A, B, or C. Each independent population was evolved for >200 generations in continuous, aerobic culture, limiting on ammonium (NH_4_^+^, supplied as (NH_4_)_2_SO_4_) as described in Materials and Methods. The hybrid strain grew robustly, achieving steady state within 10 culture generations at the target dilution rate, D = 0.16 h^−1^. Because a previous study [42] had demonstrated that *S. cerevisiae*×*S. uvarum* interspecific hybrids evolving under stress can shed one of their ancestral genomes, we performed flow cytometry on hybrids isolated at the beginning and end of our experiments. In all cases genomes were diploid, indicating that there was no large-scale loss of genome content during the experiments (data not shown); we also microscopically observed the cultures periodically during the course of the evolution and saw no evidence of asci or spores.

**Table 1:** Strains used in this study.

Strain	Description	Genotype	Strain background	Ploidy	Source
**CC6**	*S. uvarum* parent of GSY86	*MAT*a/α; *lys2-5/lys2-5*; *HO/HO*	CBS7001	Diploid	This study
**CC230**	*S. cerevisiae* parent of GSY86	*MAT*α; *ura3-52*; *ho*	S288c	Haploid	This study
**GSY86**	*S. uvarum* - *S. cerevisiae* interspecific F1 hybrid; single isolate from mass mating of CC230 with sporulated CC6; alias CC189	Suva/Scer: *MAT*a/α; *lys2-5/LYS2*; *URA3/ura3-52*; *HO/ho*	CBS7001 X S288c	Diploid interspecific hybrid	This study
**GSY2531**	T_initial_ (0 generation) GSY86 used to found Vessels A & B	Suva/Scer: *MAT*a/α; *lys2-5/LYS2*; *URA3/ura3-52*; *HO/ho*	CBS7001 X S288c	Diploid interspecific hybrid	This study
**GSY2546**	150 generation evolved GSY86 clone from Vessel A	Suva/Scer: *MAT*a/α; *lys2-5/LYS2*; *URA3/ura3-52*; *HO/ho*	CBS7001 X S288c	Diploid interspecific hybrid	This study
**GSY2547**	200 generation evolved GSY86 clone from Vessel A	Suva/Scer: *MAT*a/α; *lys2-5/LYS2*; *URA3/ura3-52*; *HO/ho*	CBS7001 X S288c	Diploid interspecific hybrid	This study
**GSY2532**	200 generation evolved GSY86 clone from Vessel A	Suva/Scer: *MAT*a/α; *lys2-5/LYS2*; *URA3/ura3-52*; *HO/ho*	CBS7001 X S288c	Diploid interspecific hybrid	This study
**GSY2548**	150 generation evolved GSY86 clone from Vessel B	Suva/Scer: *MAT*a/α; *lys2-5/LYS2*; *URA3/ura3-52*; *HO/ho*	CBS7001 X S288c	Diploid interspecific hybrid	This study
**GSY2549**	150 generation evolved GSY86 clone from Vessel B	Suva/Scer: *MAT*a/α; *lys2-5/LYS2*; *URA3/ura3-52*; *HO/ho*	CBS7001 X S288c	Diploid interspecific hybrid	This study
**GSY2550**	200 generation evolved GSY86 clone from Vessel B	Suva/Scer: *MAT*a/α; *lys2-5/LYS2*; *URA3/ura3-52*; *HO/ho*	CBS7001 X S288c	Diploid interspecific hybrid	This study
**GSY2533**	200 generation evolved GSY86 clone from Vessel B	Suva/Scer: *MAT*a/α; *lys2-5/LYS2*; *URA3/ura3-52*; *HO/ho*	CBS7001 X S288c	Diploid interspecific hybrid	This study
**GSY2534**	T_initial_ (0 generation) GSY86 used to found Vessel C	Suva/Scer: *MAT*a/α; *lys2-5/LYS2*; *URA3/ura3-52*; *HO/ho*	CBS7001 X S288c	Diploid interspecific hybrid	This study
**GSY2551**	150 generation evolved GSY86 clone from Vessel C	Suva/Scer: *MAT*a/α; *lys2-5/LYS2*; *URA3/ura3-52*; *HO/ho*	CBS7001 X S288c	Diploid interspecific hybrid	This study
**GSY2552**	200 generation evolved GSY86 clone from Vessel C	Suva/Scer: *MAT*a/α; *lys2-5/LYS2*; *URA3/ura3-52*; *HO/ho*	CBS7001 X S288c	Diploid interspecific hybrid	This study
**GSY2535**	200 generation evolved GSY86 clone from Vessel C	Suva/Scer: *MAT*a/α; *lys2-5/LYS2*; *URA3/ura3-52*; *HO/ho*	CBS7001 X S288c	Diploid interspecific hybrid	This study
**GSY1221**	GFP-marked *S. cerevisiae* parent of GSY2590	*MATa*; *ura3-52*; *YBR209W*::GFP::*ybr209w*; *ho*	S288c	Haploid	[99]
**GSY1063**	*S. uvarum* parent of GSY2590	*MAT*α; *ho::KanMX*	CBS7001	Haploid	[49]
**GSY2590**	GFP-marked *S. uvarum* - *S. cerevisiae* interspecific F1 hybrid; zygote selected from mating between GSY1221 and GSY1063	Suva/Scer: *MAT*a/α; *URA3/ura3-52*; *YBR209W/YBR209W::*GFP*::ybr209w*; *ho::KanMX/ho*	CBS7001 X S288c	Diploid interspecific hybrid	This study

### Populations of newly formed interspecific hybrids show “hybrid vigor” but limited scope for physiological improvement under nitrogen limitation

At steady state, the interspecific hybrid performed better than either of its ancestral species under aerobic ammonium limitation at 25°C. At the beginning of steady state growth (t*_-initial_*), residual ammonium was near or below detection limit (<0.01 ppm or 0.01 µg L^−1^) for both parental species and for the interspecific hybrid. Residual glucose at t*_-initial_* in diploid *S. cerevisiae*, diploid *S. uvarum* and in the interspecific hybrid was 5.0±0.34, 5.7±0.23 and 2.3±1.20, respectively (mean ± Std. Error, g L^−1^; *P* = 0.04, one-way ANOVA followed by Student-Newman-Kuels (SNK) test), while optical density at A_600_ was 0.90±0.04, 1.11±0.02 and 1.55±0.17, (mean ± Std. Error, *P*<0.01 one-way ANOVA followed by SNK test); thus for both parameters the hybrid showed superior growth performance compared to either of its parents. In three independent hybrid populations evolved for 200 generations (t_-*final*_) we detected no significant change in either residual glucose or optical density relative to the ancestral unevolved hybrid. The extent to which uptake of the limiting nutrient was enhanced could not be assessed, as ammonium concentration in the experimental populations was close to our assay detection limit at t*_-initial_* and below this limit at t_-*final*_. Based on these observations, we concluded that in a nitrogen-limited, glucose-sufficient environment, *S. cerevisiae*×*S. uvarum* interspecific hybrids had limited scope for measurable improvement in the physiological parameters we measured.

### Independently evolved hybrid clones are more fit than their common ancestor

To directly test whether individual clones from the evolved populations were more fit than their common ancestor, we performed short-term (15 generation) competitive chemostat experiments. We competed the founder *S. cerevisiae*×*S. uvarum* hybrid (GSY86) and each of three individual 200-generation evolved clones (GSY2532, GSY2533 and GSY2535, representing one isolate from each vessel; Table 1) against a fluorescently marked unevolved *S. cerevisiae*×*S. uvarum* interspecific hybrid strain (GSY2590; Table 1), under the same ammonium-limited conditions used for our long-term evolution experiments. GSY2590 is identical to the ancestral founder strain except for the presence of an integrated Green Fluorescent Protein (GFP) gene, as described in Materials and Methods. Because we observed only modest fitness differences between the founder hybrid (GSY86) and GSY2590 (competition coefficient = 1.04±0.009), we concluded that the latter could serve as a surrogate “founder” in the competitive chemostat experiments, with a 0.04 correction to the competition coefficient. We found that each of the three evolved F1 hybrids consistently outcompeted GSY2590 under continuous ammonium limitation: corrected selection coefficients for GSY2532, GSY2533 and GSY2535 were 1.15 (±0.005), 1.14 (±0.003), and 1.11 (±0.008), respectively (mean ± Std. Error). These fitness gains are statistically significant (P<0.001) and similar in magnitude to values reported for *S. cerevisiae* evolving under ammonium limitation (1.09 in [43]), but less than fitness gains reported for *S. cerevisiae* evolved under aerobic glucose limitation (1.16 to 1.60 in [44]). The scope for fitness improvement in yeast evolving at low growth rates is likely greater under aerobic glucose limitation because cells can switch from respiro-fermentative to respiratory metabolism, which greatly increases the efficiency of converting substrate to biomass [45]. Furthermore, as fungi in nature face chronic nitrogen limitation [39], [40], natural selection has likely fine-tuned mechanisms to scavenge inorganic nitrogen.

### Karyotypic evolution is evident in independent hybrid populations

**Figure 1:**
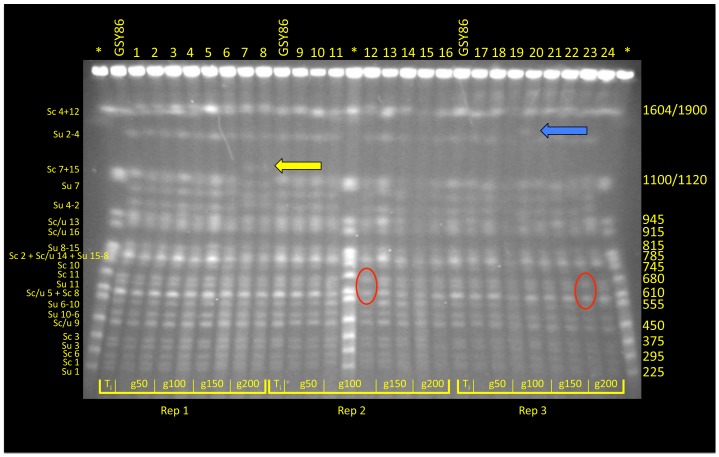
CHEF analysis of randomly selected clones from F1 hybrid evolution experiments. At generations 50, 10, 150 and 200 two clones from each of three replicate populations were chosen for electrokaryotyping. Chromosome length variants were evident in all populations: in vessel A (Replicate 1) a yellow arrow indicates an accretion in the size of one chromosome associated with the *S. cerevisiae* Chromosome 7+15 doublet; in vessels B and C (Replicates 2 and 3), red ovals denote absence of a 650 Kb band corresponding to *S. uvarum* Chromosome 11; size variation was also noted in *S. uvarum* Chromosome 2–4, denoted by a blue arrow. Asterisk* = *S. cerevisiae* Yeast Chromosome PFG Marker (New England BioLabs; Ipswich, MA; # N0345S); GSY86 = Founder *S. cerevisiae*×*S. uvarum* hybrid.

For time points corresponding to generations ∼50, ∼100, ∼150, and ∼200, archived population samples from vessels A, B and C were revived from cryogenic storage and plated on YPD; for each time point two clones were selected at random for karyotype analysis using CHEF (Clamped Homogeneous Electric Fields) gel-electrophoresis; one clone from the founding (*t_-initial_*) population was also included (Figure 1). Although most isolates exhibited the parental karyotype, several variants exhibited size changes in one or two chromosomes. For example, both isolates from generation 200 of vessel A exhibited an increase in size of one of the chromosomes corresponding to the *S. cerevisiae* chromosome 7+15 doublet at 1200 Kb (Figure 1, yellow arrow). Interestingly, in both vessels B and C (inoculated independently with different starter cultures), clones isolated at generation 100 and generation 200, respectively, demonstrated absence of the ∼650 Kb band, apparently corresponding to chromosome 11 of *S. uvarum* (Figure 1, red ovals). Other karyotypes transiently appeared in the populations, such as that observed in vessel C at 100 generations involving a size increase in *S. uvarum* chromosome 2–4 at 1500 Kb (Figure 1, blue arrow), as well as multiple instances of size variation in *S. cerevisiae* and *S. uvarum* chromosome 12 (1640/1900 Kb; topmost chromosomal band seen in Figure 1). This last observation may reflect variation in copy number of tandemly-arrayed ribosomal DNA repeats on chromosome 12, as this region of the yeast genome is known to be labile [46].

### Array-Comparative Genomic Hybridization (aCGH) reveals additional changes in genome content

CHEF analyses clearly demonstrated genome malleability in interspecific hybrids evolving under continuous nitrogen limitation. However, while CHEF analysis reveals the phenomenon of malleability, and serves as a screen to identify interesting karyotypes, it tell us little about the underlying architectural changes, and nothing at all about the molecular mechanisms that might be at play. To further investigate the evolved clones, we used aCGH to assay whole genome copy number variation arising from non-copy-neutral changes such as deletions, amplifications and non-reciprocal translocations. aCGH profiles of evolved hybrids revealed that a small number of chromosomes had undergone rearrangement; however, the rearrangements detected using aCGH were not those detected by CHEF, indicating that the rearrangements detected by CHEF were most likely copy-neutral events.

**Figure 2:**
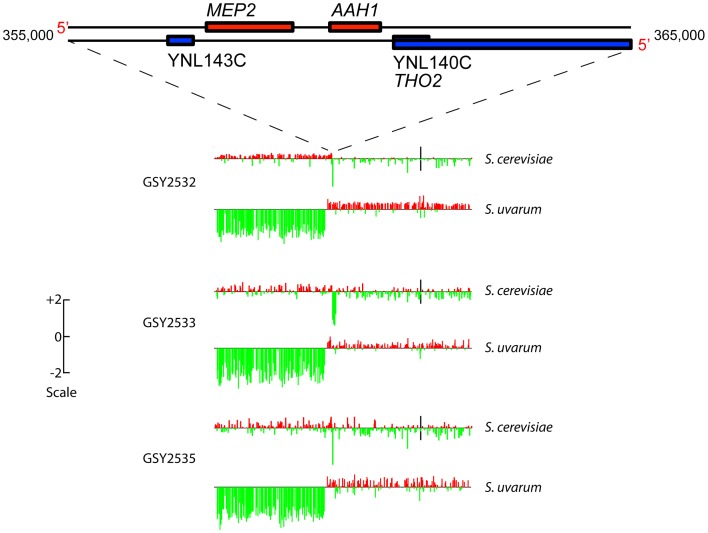
Array-Comparative Genome Hybridization (aCGH) caryoscopes of Chromosome 14 rearrangement seen in three independently-evolved F1 hybrids. Along the top is shown the gene map of a “zoomed-in” 10 Kb portion of Chromosome 14 (from coordinates 355,000 to 365,000) corresponding to the *MEP2* rearrangement region. The aCGH data are shown for 200-generation evolved clones isolated from each independent vessel: GSY2532 from Vessel A, GSY2533 from Vessel B, and GSY2535 from Vessel C. The aCGH data shown are for the entire chromosome 14, with data shown separately for the *S. cerevisiae* and the *S. uvarum* chromosomes. Bars along the chromosome represent red∶green log ratios, with length of the bar proportional to the value of the log ratio. Red bars indicate positive log ratios (i.e., the presence and/or amplification of the genomic region corresponding to that probe) and green bars indicate negative log ratios (i.e., the depletion or deletion of the genomic region corresponding to that probe). The scale to the left indicates how the height of the bars corresponds to log ratio. Black vertical bars in the *S. cerevisiae* chromosomes correspond to their centromeres (the location of the *S. uvarum* centromeres has not been determined but is thought to be similar to that of *S. cerevisiae*).

aCGH analysis showed that clones isolated from each of two independent *t-_initial_* founding populations had the expected, non-rearranged F1 hybrid genome configuration, i.e., they contained one complete non-rearranged chromosomal set from each of the input genomes, within the limits of detection of aCGH (Figure S2). Strikingly, however, aCGH revealed that 9 out of the 10 evolved hybrid clones we examined—four of four 150-generation clones (one from Vessel A, two from Vessel B, and one from Vessel C), and five of six 200-generation clones (2 each from Vessels A, B, and C)—contained a distinctive and apparently identical, or extremely similar, rearrangement on chromosome 14, whereby fully half of the *S. uvarum* chromosome 14 (much of the distal portion of the left arm) was replaced with the corresponding region of the *S. cerevisiae* chromosome 14 (Figure S2). This appeared in all cases to be a “non-reciprocal” translocation event, resulting in increased copy number of the distal left portion of the *S. cerevisiae* chromosome 14, with the concomitant deletion of the corresponding *S. uvarum* chromosome 14 region (detailed aCGH results for the chromosome 14 region are shown for three 200-generation clones, one from each vessel, in Figure 2). Because the lengths of the translocated regions of the two chromosome 14 s are roughly equivalent between these species, we would not expect to see in these clones any change in chromosome 14 mobility by CHEF, and in fact, none was seen (see Figure 1).

Because this rearrangement is seen in clones from all three vessels, it must have arisen independently. The fact that the chromosome 14 rearrangement occurred independently and is seen in a large majority of evolved clones examined suggests that it is adaptive under inorganic nitrogen limitation; indeed, as shown below, the rearrangement always occurs precisely within the *MEP2* gene (YNL142W), which encodes the high-affinity, low capacity ammonium permease in *Saccharomyces* [47], [48]. The *MEP2* gene is found in both the *S. uvarum* and *S. cerevisiae* genomes, in the same (syntenic) position on each genome's chromosome 14, sharing 85% DNA sequence identity. In addition to the *MEP2* rearrangement, a few additional rearrangements resulting in copy number variation—including deletions of ∼15 to ∼50 kb occurring on chromosomes 5, 12, and 15 of *S. cerevisiae* and chromosome 9 of *S. uvarum*, plus a probable extra copy of *S. cerevisiae* chromosome 12 in Vessel B clones—were evident among some of the evolved clones, but none of these were shared across vessels (Figure S2).

### Sequencing of evolved clones' chromosome 14 junction regions

We designed primers well outside the chromosome 14 fusion junctions detected by aCGH in the evolved clones (Table S1) to PCR-amplify the junction-containing regions of the three 200-generation evolved clones whose aCGH results are shown in Figure 2; these are clones GSY2532, GSY2533, and GSY2535, coming from Vessels A, B, and C, respectively (Table 1). Sanger sequencing of these PCR products revealed that the junction breakpoints of the rearrangement differed among clones (Figure S3A), indicating that despite appearing almost identical by aCGH, the rearrangements were indeed independent, as expected since the clones arose in three separate vessels. The junction sites for these three clones were all located within the coding sequence of the *MEP2* gene and in all three cases the gene remained in-frame. For GSY2532 and GSY2535 the junctions result in a predicted fusion protein with the N-terminal one-third (approximately) of the protein coming from *S. cerevisiae* and the C-terminal two-thirds from *S. uvarum*; for GSY2533, these proportions are swapped (Figure S3B). The *S. cerevisiae* and *S. uvarum* Mep2 proteins are each 499 amino acids long, with 17 amino acid differences between them; each of the three predicted Mep2 fusion proteins has a novel predicted protein sequence derived from the combination of the *S. cerevisiae* and *S. uvarum MEP2* genes.

### Whole-genome sequencing of ancestral and evolved clones

**Figure 3:**
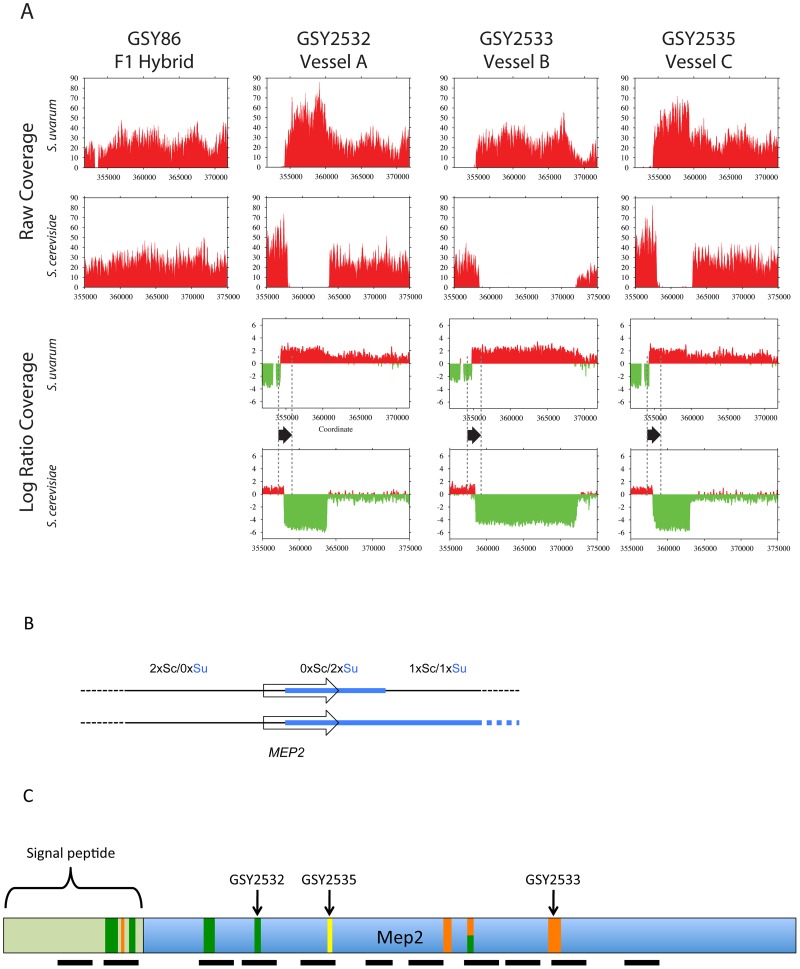
Further analysis of *MEP2* gene fusion rearrangements. (A) Depth of coverage plots from whole genome sequence of three independently evolved F1 hybrids. All panels show read coverage data from whole genome sequencing for the 20 kb region surrounding the *MEP2* gene on chromosome 14 for both *S. cerevisiae* and *S. uvarum* (with chromosomal coordinates shown below), such that the start codon of *MEP2* is precisely aligned between the two species. GSY86 is the ancestral unevolved F1 hybrid and GSY2532, GSY2533 and GSY2535 are 200-generation evolved clones isolated from Vessels A, B, and C, respectively. The lower plots show ancestor-normalized log-ratio values for the evolved clones, with the start and stop codon boundaries of the *MEP2* gene shown as dotted lines and the gene itself shown as a black arrow. In GSY86 there was no coverage for a small section of the *S. uvarum* genome upstream of the MEP2 start codon (upper left plot); this region coincides with the junction of two contigs in the original *S. uvarum* assembly. Based on our Sanger sequencing of the region, the lack of coverage likely corresponds to a small misassembly in the sequence we used as the reference genome. To avoid a divide by zero error, no log ratio data were calculated for this region, yielding a small “gap” in the *S. uvarum* log ratio plots. (B) Structure of *MEP2* region rearrangement found in three independently evolved F1 hybrids by whole genome sequencing. Schematic representation of the genome configuration of the *MEP2* fusion region for the *S. cerevisiae* and *S. uvarum* chromosome 14 s as found in the three evolved clones; thin black line = *S. cerevisiae* genomic sequences, thick light blue line = *S. uvarum* genomic sequences; arrowed box = coding region of the *MEP2* gene. Observed copy numbers for the *S. cerevisiae* (Sc) and *S. uvarum* (Su) genomic sequences across the junction region are indicated above. (C) Locations of *MEP2* gene fusion junctions found by targeted sequencing in multiple clones from independent evolved populations. The entire Mep2 protein is shown to scale, with signal peptide shown as labeled light green box on left; the 11 transmembrane domains are shown as black horizontal bars below. Vertical bars show the location of all characterized junctions; the width of each bar is to scale for the region of shared identity between the two species found at the particular junction. Green vertical bars show junctions found in Vessel A, orange for Vessel B, and yellow for Vessel C (note that multiple clones from Vessels A and B were characterized, compared with only one clone from Vessel C). The half green-half orange bar represents a junction found in both Vessels A and B. Junction positions of the whole-genome-sequenced clones GSY2532, 2533 and 2535 are indicated.

To further elucidate genomic changes that occurred during evolution of the interspecific F1 hybrids, we performed Illumina whole genome sequencing on the three independent 200-generation evolved clones containing *MEP2* fusion genes, whose junctions we had sequenced as described above (GSY2532, 2533, 2535), and also on the ancestral clone used to found the three replicate vessels (GSY86). Read depths across the *MEP2* regions indicated that as expected for the ancestral GSY86 clone, there were no rearrangements resulting in copy number changes in either genome's *MEP2* region (Figure 3A).

In contrast, and confirming our aCGH results, we detected large-scale copy number changes in the *MEP2* region for each of the three evolved clones (Figure 3A). Surprisingly, however, we observed from our whole genome sequencing that the architecture of the genome rearrangement was more complex than we had predicted by aCGH. Instead of a simple translocation (and/or “breakage-induced replication” event) to yield one *S. cerevisiae – S. uvarum* chimaeric chromosome (with the junction located within the *MEP2* gene) and one intact *S. cerevisiae* chromosome, in all three cases the expected *S. cerevisiae – S. uvarum* chimaeric chromosome was present, but there was an additional rearrangement on the *S. cerevisiae* chromosome (Figure 3A,B). This additional event resulted in a complete deletion of 5 to 15 kb of the *S. cerevisiae* chromosome, the region instead being precisely replaced with the corresponding *S. uvarum* chromosomal region within an otherwise intact *S. cerevisiae* chromosome; this is an event that can be considered to be the equivalent of an “introgression” of the *S. uvarum* genome into the *S. cerevisiae* chromosome (Figure 3B). In each case the distal junction on the *S. cerevisiae* chromosome occurred within the *MEP2* gene, with exactly the same junction as that found in the partner *S. cerevisiae – S. uvarum* chimaeric chromosome (note: this is why our Sanger sequencing of PCR products described above gave readable sequences). In all cases the junction found by whole genome sequencing matched exactly the junction we had found by Sanger sequencing. The proximal junction was always well “downstream” from the *MEP2* gene and varied for each clone, occurring anywhere from ∼5 Kb (GSY2532 and GSY2533; within *THO2*) to 15 kb (GSY2535; near *FPR2*) toward the centromere (Figure 3A). The most interesting outcome of this additional rearrangement within the *S. cerevisiae* chromosome is that each of the evolved clones contains two copies of identical *MEP2* fusion genes (with junctions as shown in Figure S3A and S3B), and no copies of either the *S. cerevisiae* or the *S. uvarum* endogenous (“wild-type”) *MEP2* genes. Analysis of the whole genome sequences for shared SNPs (and/or shared SNP-containing genes) revealed that no such shared mutations existed among the evolved clones (Table S2).

### Multiple *MEP2* gene fusion alleles coexist in the same population, and *MEP2* fusions coexist with *S. uvarum MEP2* in the same genome

Because we saw *MEP2* rearrangement events occurring on both the *S. uvarum* and *S. cerevisiae* chromosomes of the evolved clones, we wished to know if the rearrangements occurred in a single concerted step, or whether a sequential multi-step process led to the final configuration. We therefore performed diagnostic PCRs on 12 single colony isolates from evolved populations corresponding to 0, ∼50, ∼100, ∼150 and ∼200 generations, from Vessels A and B, for a total of 120 isolates (12 per time point, 60 per vessel). We used 4 PCR primer combinations for each clone, using primer combinations (Table S1) specific for the *S. cerevisiae MEP2* gene, the *S. uvarum MEP2* gene, the *S. cerevisiae-S. uvarum* fusion *MEP2* gene (found in evolved clones), or the *S. uvarum-S. cerevisiae* “reverse-fusion” *MEP2* gene (not found in the evolved clones described above that were examined by aCGH and/or sequencing). Almost all clones from generations 0 and 50, as expected for an un-rearranged (“ancestral”) hybrid, showed the coexistence of the *S. cerevisiae MEP2* gene and the *S. uvarum MEP2* gene, with no evidence of a *MEP2* fusion gene (Figure S4A). We further found that the *MEP2 S. cerevisiae-S. uvarum* fusion gene appeared in both vessels starting at 100 generations and persisted through to the 200-generation time point (Figure S4A). At 100 generations, in both vessels, less than 20% of the clones contained the ancestral un-rearranged *MEP2* genes; instead most clones contained the *MEP2* fusion gene either alone (presumably in two copies as seen in GSY2532, 2533, and 2535), or the fusion gene in conjunction with the *S. uvarum*-only *MEP2* gene. By the 150 and 200-generation time points, the *MEP2* fusion gene alone was predominant. In these later time points, there also appeared clones containing only the *S. cerevisiae MEP2* gene or only the *S. uvarum MEP2* gene, without the presence of the *MEP2* fusion gene (Figure S4A). Interestingly, although we observed the *MEP2* fusion gene in conjunction with the *S. uvarum MEP2* gene, we never observed the *S. cerevisiae MEP2* gene occurring with the *MEP2* fusion gene. Finally, the “reverse-fusion” *MEP2* gene was not found in any of the 120 clones.

We Sanger-sequenced the PCR products corresponding to the *MEP2* fusion gene from all clones yielding such PCR products. We found that there were several additional *MEP2* fusion junctions present in the evolved clones of both vessels, with junctions differing from those found in the three clones (GSY2532, 2533, and 2535) we had previously characterized by Sanger and whole genome sequencing. As seen in Figure 3C and Figure S4B, in addition to the junctions found for GSY2532 (Vessel A) and GSY2533 (Vessel B), four additional distinct and separate novel *MEP2* fusion junctions were found in Vessel A clones, and a further three distinct and separate novel *MEP2* fusion junctions were found in Vessel B, for an observed total of nine different *MEP2* gene fusion junctions (including that of GSY2535); in all cases, the junctions occurred within the *MEP2* coding sequence and were in-frame.

### qRT–PCR assays reveal only slight difference in transcription levels of *MEP2* genes from each genome within a hybrid

Because the evolved fusion genes have the *S. cerevisiae MEP2* promoter, we hypothesized that the *MEP2* gene fusion events may have been selected because that promoter might result in higher transcript levels. We thus performed qRT-PCR reactions for each genome's version of the *MEP2* gene on the founding ancestor GSY86, assaying (in triplicate) two independent biological replicates of GSY86 that had been grown to steady state in the same nitrogen-limited media and chemostats used for the original evolutions. We determined that the *S. cerevisiae* genome's copy of the *MEP2* gene is indeed expressed at a somewhat higher level than the *S. uvarum* copy, by almost 2-fold, supporting our hypothesis (Figure S5; raw and normalized data given in Table S4). However, when we determined the expression of the fusion gene in an evolved clone (GSY2532), it appeared to produce less transcript per locus than either the *S. cerevisiae* or *S. uvarum* genes did in the founding hybrid (Figure S5, Table S4; note, in the evolved clone, the transcript quantified by qPCR is produced from 2 fusion loci, so the amount per locus is less). The mechanistic basis for *MEP2* fusion genes' adaptive advantage is therefore more complex than increased expression, and may relate instead to changes in protein structure that increase the novel permeases' catalytic efficiency, decrease their *K_m_* for ammonium, and/or alter their activity as nutrient signaling molecules.

## Discussion

### Fungal genome architecture varies both in nature and in the laboratory

Laboratory strains of *S. cerevisiae* are the best-studied group of fungi in terms of their genome structure. However, even within this relatively homogeneous group, strains differ widely in rates of mitotic chromosome loss and levels of chromosome-length polymorphism [49]–[51]. Furthermore, mitotic genome instability in *S. cerevisiae* has been shown to be evolutionarily significant in the laboratory [52]–[54], in wine fermentation [24], [55] and in biomass conversion to fuel ethanol [56]. A large amount of standing genomic variation (e.g., ploidy differences, transposon copy number, and chromosome length polymorphism) is found among *Saccharomyces* isolates collected from natural and industrial settings (e.g. [57]–[59]), reinforcing the view that genomic plasticity may be evolutionarily important in diverse settings (see [10], [24] for reviews). Of special relevance to our study is the discovery that this variation very often takes the form of mosaic genomes that result from natural interspecific hybridization events [20], [28], [60]–[63]. Mosaic genomes arising from interspecific hybridization have been discovered in other yeasts. For example, *Pichia sorbitophila* appears to have arisen in recent centuries via allopolyploidization between two species affiliated with the genus *Millerozyma* [64]. Resolution of the initial hybridization event has produced 7 chromosome pairs that are either completely homozygous, completely heterozygous or mosaics. In mosaic chromosomes, breakpoints between homozygous and heterozygous regions can occur in protein coding genes [64], though with unknown phenotypic consequences. While the foregoing example provides an interesting snapshot of a recent hybridization event, no published study to date has explored genome dynamic changes that occur as experimentally-created interspecific hybrids evolve.

### Interspecific hybrids evolved under limiting nitrogen exhibit recurrent independent rearrangements of the *MEP2* ammonium permease gene

Using CHEF and aCGH analysis we were able to detect chromosomal loss and/or size changes, large indels, and non-reciprocal translocations in evolving interspecific F1 hybrids. Overall, however, the frequency with which we observed genomic rearrangements in hybrids evolving under nitrogen limitation was considerably less than that reported for *S. cerevisiae* evolving under glucose limitation [53]. Further, very few large-scale genomic changes were observed by CHEF and aCGH analysis when the diploid parental species themselves, *S. cerevisiae* and *S. uvarum*, were evolved under nitrogen limitation (Dunn, Piotrowski *et al.* in prep.). However, for a large number of evolved interspecific hybrid clones we observed a distinctive recurrent rearrangement, involving both parental genomes at the locus encoding high affinity ammonium permease, which we first observed by aCGH and then confirmed by Sanger and whole genome sequencing. These recurrent *MEP2* rearrangements in *S. cerevisiae*×*S. uvarum* hybrids provide an interesting contrast with the results of experimentally evolving haploid *S. cerevisiae* under different types of limiting nitrogen. There, recurrent rearrangements were observed at the *GAP1* locus, which encodes for the general amino acid permease [43]. A single homologous recombination event was seen to produce two different alleles: *GAP1^extrachromosomal circle^* or *gap1Δ*; the former being associated with higher fitness in clones adapted to L-glutamine and L-glutamate, the latter with higher fitness in clones adapted to urea, allantoin, and ammonium. Owing to differences in genome content, hybrid interspecific diploids are able to explore adaptive possibilities not open to haploid *S. cerevisiae*.

While we failed to detect any mutations or rearrangements that inactivate *GAP1* in any of the ammonium-adapted clones that we sequenced, our observations are remarkably similar to what occurs when either haploid or diploid *S. cerevisiae* is evolved under sulfur limitation, where a recurrent rearrangement, resulting in gene amplification, has been observed at the *SUL1* locus which encodes a high affinity sulfate permease of the SulP anion transporter family [65]. In our experiments, the recurrent event is a complex rearrangement at the *MEP2* locus, which encodes a high affinity ammonium permease. This rearrangement yields a genome containing two copies of a fusion *MEP2* gene with the 5′ portion derived from *S. cerevisiae* and the 3′ portion from *S. uvarum*. It is likely that this rearrangement is adaptive under N-limitation, due to both its high allele frequency and the fact that it was selected for independently multiple times. As expected, when tested in direct head-to-head competition experiments, each of three independently-evolved evolved clones having the characteristic *MEP2* rearrangement showed significant fitness increases relative to an unevolved ancestral clone. We have not yet shown that the presence of *only* the two *MEP2* fusion genes is necessary and sufficient to confer this selective advantage within the context of an otherwise unevolved hybrid. Nevertheless, whole genome sequencing of these three evolved clones revealed no shared SNPs or rearrangements in other genes, suggesting that the recurrent *MEP2* rearrangement is a key shared adaptive innovation in these evolutions, an innovation unavailable to either parent alone.

### Fusion genes as sources of evolutionary innovation

A number of recent studies suggest that gene fusions may contribute to the evolution of novel functions (reviewed in [66]). Because new folding structures could quickly produce traits unattainable by point mutation alone, fusion genes could be potent drivers of adaptive change [67], and indeed, *in vitro* generation of fusion genes has been directly shown to create novel enzymatic functions [68]. Fusion genes have been discovered in many organisms, and even play an important role in the initial steps of tumorigenesis [69]. Examining hybrid lager yeast, Usher and Bond recently described a fusion gene formed by recombination between homoeologous chromosomes of *S. cerevisiae* and *S. eubayanus* [21]. The result, a chimaeric gene for *GPH1*, which encodes for glycogen phosphorylase, fails to produce mature mRNA because of a frameshift in its coding sequence; loss-of-function at *GPH1* leads to a glycogen phenotype typical of haploid cells. In contrast, the chimaeric genes that we discovered in the course of evolving yeast hybrids are always formed by in-frame fusions of the 5′ end of the *S. cerevisiae MEP2* coding sequence to the 3′ end of the *S. uvarum MEP2* coding sequence.

### What is the nature of the fitness advantage in the evolved clones?

Chemostat theory [70], [71] predicts that when cells evolve under nutrient limitation, adaptive genotypes arise as a result of either increased efficiency of nutrient use or increased capacity for assimilating the limiting nutrient. Previous studies have shown that in *S. cerevisiae* evolving under glucose limitation both mechanisms come into play [45], [53], [72], resulting in increased yield biomass and diminished concentrations of residual substrate at steady state. To test whether this was also the case in our experiments, we grew to steady state under ammonium limitation single-colony isolates of the ancestral hybrid and the three evolved clones that we sequenced. We found that in all cases residual ammonium was near or below detection limits (0.01 ppm), which follows from the very low K_m_ of ammonium permease (1–2 µmolar; [73]) and the high velocity of ammonium uptake (<0.25 µmolar per second at D = 0.1 h^−1^), under glucose-sufficient conditions. We found that culture density and residual glucose concentrations for ancestral and evolved strains were also not statistically different (though interestingly, GSY2532 and GSY2535 produced a small but significantly greater amount of dry weight biomass than their ancestor, see Table S3, *P* = 0.002). These biochemical results, are, as for the genomic results, again reminiscent of those obtained when *S. cerevisiae* is experimentally evolved under inorganic sulfur limitation [65]. Evolved sulfur-limited populations show very modest increases in cell biomass, compared to evolved glucose and phosphate-limited populations, even though *SUL1*, which encodes a high-affinity sulfate transporter, is amplified in multiple independent evolutions, and even though this mutation demonstrably increases fitness when crossed into an unevolved wild-type background. Gresham *et al.* [65] conclude that the scope for metabolic innovation in inorganic sulfur metabolism is constrained, in this case by the small contribution of sulfur to cell biomass, relative to that of glucose or phosphate, and that this constraint results in the repeated evolution of a rearrangement resulting in *SUL1* amplification. Our nitrogen-limitation results suggest metabolic constraints of a similar nature, which may be driven in part by the fact that fungi as a group are chronically nitrogen limited and have likely been under strong selection to acquire the capacity to scavenge this element to extremely low levels.

Our qRT-PCR results from the unevolved hybrid show a relatively modest two-fold difference in expression of the *MEP2* gene from each of the genomes present in the unevolved hybrid, with *S. cerevisiae* the higher expressed gene of the two. This would seem to indicate that the unidirectional nature of the fusion gene rearrangement, whereby we always observe the *S. cerevisiae* promoter and 5′ end of the gene fused to the 3′ end of the *S. uvarum* gene, arises simply to increase overall transcript levels of the *MEP2* gene. However, in an evolved clone, we surprisingly discovered that the fusion gene produces slightly lower transcript levels per locus than does either the wild-type *S. cerevisiae* or *S. uvarum* locus in the progenitor hybrid. It is unclear exactly what this finding means – possibly that transcription from the *MEP2* gene is governed by a feedback mechanism that reduces its transcription.

It may be that the actual fusion proteins themselves, despite the few amino acid differences they show relative to the two parental genes (Figure S3B), provide an adaptive advantage. Possibly the novel chimaeric ammonium permeases differ from their ancestors in having a lower *K_m_*, which would lead to lower residual ammonium levels, and/or a higher *k_cat_*, which would result in greater overall uptake velocity. Alternatively, because in *S. cerevisiae* the Mep2 protein forms multimeric complexes in the plasma membrane [74], it may be of adaptive benefit to hybrids to produce Mep2 proteins that contain only *S. cerevisiae* N-termini and/or only *S. uvarum* C-termini; this could possibly result in better oligomerization for improved transport function and/or prevent dominant negative interactions between the two species' proteins. Indeed, dominant negative interactions have previously been noted between different alleles of the closely-related Mep1 and Mep3 proteins in yeast [75]. In this regard it is also provocative that we did not observe the coexistence of the *MEP2* fusion gene with the *S. cerevisiae MEP2* gene in any of the 120 clones that we genotyped, although we did see coexistence of the *MEP2* fusion gene with the *S. uvarum MEP2* gene; this may be evidence for dominant negative interactions between the *MEP2* fusion gene and the *S. cerevisiae MEP2* gene.

### A novel mechanism to generate introgressions rapidly

**Figure 4:**
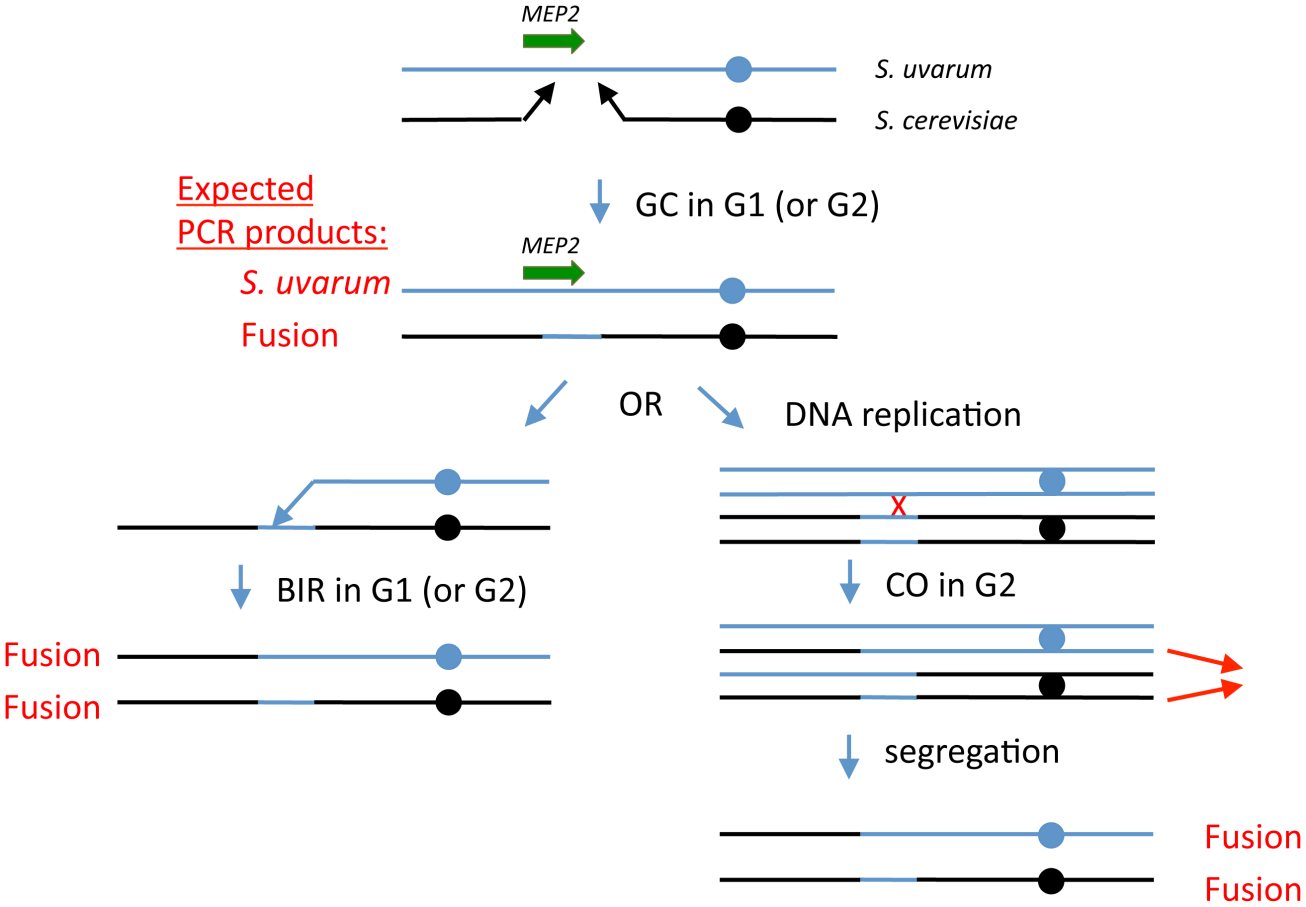
Model for generation of *MEP2* gene fusion rearrangements found evolved hybrids. Black lines represent *S. cerevisiae* genomic sequences and blue lines represent *S. uvarum* genomic sequences; the *MEP2* gene is shown as a green arrow. Expected PCR products from cells at any stable (i.e., able to perform mitosis and propagate) stage of the model are indicated in red text. First, we propose that during G1 (i.e., prior to DNA replication), in an ancestral F1 interspecific hybrid cell, a double strand break occurs in *S. cerevisiae* chromosome 14, either in the coding sequence of *MEP2* itself or somewhere within a few kb downstream of the gene. Resection of the broken ends leads to a gap in the *S. cerevisiae* chromosome. Each of the broken ends then finds a homologous region within the *S. uvarum* chromosome that allows repair of the gap, substituting the *S. uvarum* chromosomal sequences for the lost *S. cerevisiae* chromosomal sequences; this is analogous to a gene conversion (GC) event without an associated crossover. At this point, the cell's genome is stable and can propagate; this cell will contain a *S. uvarum MEP2* gene plus a *S. cerevisiae* - *S. uvarum* fusion *MEP2* gene. After this, we propose a second recombination event, either a break-induced replication (BIR) event in a G1 cell, involving the *S. uvarum* chromosome (shown on the left side of the figure), or a mitotic crossover (CO) event in a G2 cell (right side), followed by co-segregation of the two chromatids shown by thin red arrows. In both cases, the final genomic configuration of the evolved clones—containing two copies of the *MEP2* fusion gene, and no copies of either parental *MEP2* gene, with one chimaeric chromosome and one “introgressed” chromosome—is achieved.

Based on our genotyping results shown in Figure S4A, we believe that a two-step recombination event such as that shown in Figure 4 occurred between *S. cerevisiae* and *S. uvarum* chromosomes to generate the rearrangements seen in the evolved clones. We presume the event began with a double-strand break (DSB) in or near the *S. cerevisiae MEP2* gene, followed by some amount of resection of the sequences surrounding the break (as described in [76], [77]). Strand invasion into a homologous region of the *S. uvarum* chromosome would have then been followed by repair of the resected sequences using the *S. uvarum* chromosome as a template, creating a gene conversion event with resultant loss of heterozygosity (LOH) (top portion of Figure 4). At the end of the first event, the *S. uvarum* chromosome would have been intact, while the other resultant chromosome would still be almost completely composed of *S. cerevisiae* sequences, aside from several Kb of *S. uvarum* genome precisely substituted at the *MEP2* region (the exact Kb of *S. uvarum* sequences would depend on the amount of resection on either side of the DSB). Subsequently, either a DSB in the *S. uvarum* chromosome within the shared *MEP2* gene region, followed by break-induced replication (BIR), or alternatively, a mitotic crossover event in G2 (left and right lower portions, respectively, of Figure 4) would have led to the final evolved genome configuration of two fusion *MEP2* genes, sharing an identical fusion junction. Such gene conversion and BIR mechanisms have been previously well-documented and described in detail for yeast and many other organisms (see [78], [79] for reviews). We believe that a two-step process brought about the final evolved clone configuration, because some of the isolates from the ∼100 generation time-points in two independent populations (Figure 3A) showed the coexistence of the fusion *MEP2* gene with the *S. uvarum MEP2* gene (as for the intermediate shown in the first step of Figure 4). We further believe that genetic information was always transferred unidirectionally by a gene conversion event from *S. uvarum* to *S. cerevisiae*, because we never observed coexistence of the fusion *MEP2* gene with the intact *S. cerevisiae MEP2* gene (as depicted in Figure S6A and S6B), and we never detected the *S. uvarum* - *S. cerevisiae* “reverse” fusion (as depicted in Figure S6B). These findings suggest that the alternative models shown in Figure S6A and S6B are unlikely. Interestingly, this same type of event, leading to a virtually identical rearrangement configuration, has been seen in chromosomes of mouse cell lines lacking the Bloom syndrome helicase [80]; other similar, but not identical, patterns of rearrangement have been seen in yeast using plasmid-based “chromosome fragmentation vectors” and are thought to arise from template switching [81].

Introgression—infiltration of the “germplasm” of one species into that of another—occurs widely [16] and may produce extreme transgressive traits [32], which can drive rapid evolution and even speciation [30], [31]. Horizontal gene transfer, long known to be an engine of biodiversity in prokaryotes, has also been observed between eukaryotes [82], and in yeasts has recently been shown to be a mechanism by which “germplasm infiltration” can rapidly occur. Galeote *et al.* reported variable integration of a 17 kb ARS-containing *Zygosaccharomyces bailii* genome segment into dozens of *S. cerevisiae* wine strains [83]; this *Z. bailii* insertion was first discovered by whole genome sequencing of a wine strain [84]. The organization of *Z. bailii* insertions and the conspicuous absence of sequence similarity at breakpoints suggest they replicate via an extrachromosomal circular intermediate and insert via nonhomologous recombination. By contrast, introgression that arises via interspecific hybridization is currently thought to occur slowly, requiring repeated backcrossing with one of the parental species [15]. However, the structure of the *MEP2* rearrangements we have discovered suggests a mechanism by which introgressive hybridization can occur rapidly. In each case, one of the rearranged chromosomes consists almost exclusively of *S. cerevisiae* sequences except for a precise replacement of several Kb with *S. uvarum* sequences. Diploid *MEP2* fusion hybrids that undergo meiosis may produce a small number of spores that contain a haploid complement of only *S. cerevisiae* chromosomes including the one *S. cerevisiae* chromosome with the *S. uvarum MEP2* region. Alternatively, loss of the *S. uvarum* chromosomes from the *MEP2* fusion hybrid, similar to what has been described before for interspecific hybrids undergoing selection [42], could result in an “*S. cerevisiae*” strain containing just the introgressed *S. uvarum MEP2* region. These scenarios do not require repeated backcrosses to one of the parents, and open up the possibility for rapidly evolving adaptive innovations forbidden to either parental species.

## Materials and Methods

### Strains

The *S. cerevisiae* parental strain is a derivative of laboratory strain S288C (strain “CC230”; *MAT*α; *ura*3-52; *ho*), while the *S. uvarum* parental strain is derived from strain CBS7001 (strain “CC6”; *MAT*a/*MAT*α; *lys*2-5/*lys*2-5; *HO*/*HO*; see Table 1 for complete list of strains used in this study). Their F1 interspecific hybrid, GSY86, was obtained by mass-mating CC230 with mass-sporulated CC6 and selecting for prototrophy. Individual evolved clones that were further studied are also shown in Table 1.

### Chemostat media and culture conditions

The “Delft” nitrogen-limiting medium used for batch and chemostat cultures was based on that described by Boer *et al.* [85] as follows: the basal nitrogen-limiting medium (“basal salts”) consisted of the following components *per liter*: 0.15 g (NH_4_)_2_SO_4_, 5.3 g K_2_SO_4_, 3.0 g KH_2_PO_4_, and 0.5 g MgSO_4_.7H_2_O, to which was added 1× vitamins and 1× trace metals (both as in [86]), as well as 0.02 g uracil, 0.03 g lysine, 0.06 g leucine and 9 g glucose. Strains were grown at 25°C in 500 mL fermenters (INFORS AG) with a working volume of 300 mL. Impeller speed was set to 300 rpm; airflow to 10 L h^−1^; the target dilution rate was 0.16 h^−1^.

### Founding and sampling of the experimental populations

The founder F1 hybrid was first grown overnight in 2 mL of YPD-1% glucose, whereupon 500 µL of this culture was transferred into 25 mL Delft nitrogen-limiting media and grown overnight at 25°C. Three mL of this culture were sterilely transferred into 27 mL of sterile glucose, vitamins, metals, uracil, leucine, and lysine at the prescribed concentrations, and the suspension added by positive pressure to an INFORS vessel containing 270 mL of autoclaved basal salts. Three separate fermenters (Vessels A, B, and C) were inoculated in this manner. Populations were sampled every 48 h (∼10 generations). Two and one-half mL of cell suspension were withdrawn from the chemostat vessels and apportioned as follows: (i) 500 µL were added to 500 µL sterile 30% glycerol, then archived in duplicate at −80°C; (ii) 1 mL of cells were sterile-filtered through a 0.45 µm in-line filter and retained for assay of extracellular metabolites, (iii) 100 µL were diluted 9∶1 in glass-distilled water, and optical density measured at 600 nm using a Spectronic Biomate 3 spectrophotometer. Every 50 generations, archived populations were streaked onto YPD agar, and a random subset karyotyped by CHEF gel electrophoresis, as described below. A subset of these clones was further analyzed for changes in genome architecture by aCGH and by Illumina whole genome sequencing, as described below.

### Metabolite and biomass assays

Residual *glucose* was assayed spectrophotometrically on cell-free filtrate using R-Biopharm assay Kit #716251 (R-Biopharm, Darmstadt, Germany). Residual *ammonium* was determined by a modified version of the Berthelot reaction [87], scaled down for 96-well format. Biomass was estimated by filtering 50 mL of chemostat culture onto tared 0.45 µm nylon filters, and drying filters in a desiccator at 37°C for 48 hrs. To test for statistically significant differences in growth and residual metabolites between experimental populations from the first steady-state (*t-_initial_*, 10 generations) and the last (*t_-final_* at ∼200 generations) time points, and to test physiological data obtained by growing single clones to steady state, we used ANOVA followed by a Student-Newman-Kuels multiple comparison test. All statistics were calculated with Sigma Plot 11 (Systat Software, San Jose, CA).

### Analysis of yeast karyotypes

CHEF analysis was performed on two randomly-chosen single colonies isolated on YPD agar from frozen glycerol stocks of ∼50, ∼100, ∼150, and ∼200 generation population samples, from each of the three independent experimental populations. Analyses were performed essentially as described [88], [89].

### Array-based Comparative Genomic Hybridization (aCGH)

A complete description of the 2-species (*S. cerevisiae* and *S. uvarum*) array design is given in [62]; arrays were manufactured by Agilent and contain ∼5500 oligonucleotide species-specific probes per species, approximately evenly spaced across each genome. Genomic DNA from ancestral and evolved clones was prepared using Zymo Research YeaStar columns according to the manufacturer's recommendations, and then digested with *Hae*III. We then labeled 350 ng of this DNA with Cy5 (red). We similarly labeled, but instead with Cy3 (green), the same amount of reference DNA, which consisted of an equimolar mix of sheared genomic DNA from *S. uvarum* (CBS7001) and from *S. cerevisiae* (S288c). The labeled experimental and reference DNAs were then mixed together and hybridized to the 2-species microarrays as described [62]. Microarray data have been deposited in the Gene Expression Omnibus (GEO) repository [http://www.ncbi.nlm.nih.gov/geo/] under accession GSE18060. The Caryoscope program [90] was used to view microarray data in a genomic context.

### Colony–PCR assay of multiple clones from population samples

Single-colony isolates were obtained by plating onto YPD agar small aliquots of the populations corresponding to 0, ∼50, ∼100, ∼150 and ∼200 generations, from Vessels A and B. Twelve isolates per time point, per vessel (60 isolates total per vessel) were subjected to colony lysis and subsequent PCR using primer combinations (Table S1) specific for the: (1) *S. cerevisiae MEP2* gene, (2) *S. uvarum MEP2* gene, (3) *S. cerevisiae-S. uvarum* fusion *MEP2* gene found in evolved clones, and (4) *S. uvarum-S. cerevisiae* “reverse-fusion” *MEP2* gene. PCR products that arose from the *S. cerevisiae-S. uvarum MEP2* fusion gene-specific PCRs were Sanger-sequenced using the sequencing primers shown in Table S1.

### Whole-genome DNA sequencing and analysis

DNA isolation was performed, using Qiagen G-100 genomic-tip columns as described by the manufacturer, from strains GSY86, GSY2532, GSY2533, and GSY2535. The DNA was then used to prepare libraries for Illumina sequencing as described [91], using barcoded adaptors for multiplexed paired-end sequencing. Flow cells for the Illumina HiSeq 2000 platform were prepared according to manufacturer's instructions and sequencing was performed for 100 cycles for each of paired-end reads. Read data has been deposited at the NCBI under BioProject PRJNA172024. Reads were mapped using BWA [92] to a combined *S. cerevisiae* – *S. uvarum* reference genome (*S. cerevisiae* genome downloaded from http://www.yeastgenome.org on Feb 24, 2011, plus *S. uvarum* genome assembly downloaded from http://saccharomycessensustricto.org May 26, 2011). SNPs were identified using the GATK [93], [94]. No subsequent hard-filtering of identified SNPs was performed; instead, SNPs present in the unevolved GSY86 ancestor were discarded from the analysis, and the remaining SNPs present in the evolved clones were manually inspected using Samtools tview [95], with only those showing sufficient coverage and quality given further consideration; these are shown in Table S2.

### Quantitative RT–PCR

The founder interspecific hybrid GSY86 and evolved strain GSY2532 were cultured in monoculture to steady state (∼15 generations) in two independent NH_4_^+^-limited chemostats each; cultures were harvested by fast-filtration on 0.45 µm Nylon filters, frozen in liquid nitrogen, then stored at −80°C until RNA purification. Hot phenol RNA preparation was performed as described previously [96], followed by treatment with Ambion TURBO-DNAfree DNAse using manufacturer's recommendations (Life Technologies). 2 µg of total RNA were reverse transcribed using oligo dT primer and Superscript III according to the manufacturer's instructions (Invitrogen). Real-time qPCR was performed on a Bio-rad CFX96 cycler using SsoFast EvaGreen Supermix (Bio-rad). For GSY86, separate PCR reactions for detecting the *S. cerevisiae* and the *S. uvarum MEP2* transcripts were performed, using primers shown in Table S1, in technical triplicates for each of the two biological replicates. Note that the primer pairs for detecting the *S. cerevisiae* and the *S. uvarum MEP2* transcripts were first determined by PCR (using genomic DNA) to be specific for each species. Control PCR reactions (in triplicate, for each biological duplicate) for the reference *S. cerevisiae TFC1* and *S. uvarum YDR458C* genes [97] (primers shown in Table S1) were also performed. For GSY2532, qRT-PCR was performed similarly, except using primers to detect the *S. cerevisiae* - *S. uvarum MEP2* fusion transcript (see Table S1).

### Competition in chemostats

Competitive chemostat experiments were performed for the interspecific hybrid GSY86 and each of three independently-evolved strains from Vessels A, B, and C, respectively (GSY2532, GSY2533 and GSY2535). Each strain was competed pairwise (in triplicate) against a common GFP-marked F1 hybrid reference strain (GSY2590, see Table 1; it is similar to GSY86 but with the *S. cerevisiae* parent genome containing the fluorescent GFP marker inserted into the *YBR209W* locus [72]). Each strain was grown to steady state, as determined by constant optical density for ∼24 h; culture volume for the reference strain was set at 500 mL, and its competitors at 300 mL. 150 ml were removed from each competitor vessel and replaced with 150 mL of the reference strain. The competition was followed for ∼15 generations. Beginning at the time of mixing (*t*-*_zero_*), 1 mL samples of the mixed populations were collected every 8–12 h, spun at 11,000*×g* for 2 min, resuspended in 0.5 mL of 1× PBS, then stored at 4°C until FACS analysis. Flow cytometry was performed using a FACSCaliber flow cytometer (Becton Dickinson, San Jose, CA) using a 488 nm laser for excitation of GFP and signal collection using a 530-30 bypass filter. Analysis was performed using CellQuest 3.3. Selection coefficients were determined from the linear regression of ln [Test/Reference] against generations, using methods developed by [65]. We used ANOVA followed by Tukey's HSD to test for differences among competition coefficients.

## Supporting Information

Figure S1 Creating the hybrid strain for experimental evolution. Haploid *S. cerevisiae* and *S. uvarum* are mated to produce a diploid F1 hybrid. For simplicity, only 2 chromosomes are shown per cell, instead of the 16 chromosomes normally found in a haploid *Saccharomyces* cell.
(PDF)

Figure S2 Array-CGH data for F1 founders and evolved clones. Each column contains the aCGH hybridization data for a given strain (details about each strain can be found in Table 1), while each row corresponds to a probe for a chromosomal location; note that the data for each species were normalized separately. Each parental genome is shown separately, labeled at top, with probes ordered downward in chromosomal order from the left end of Chromosome I (top-most probe) to the right end of Chromosome XVI (bottom probe). The deletion of a region of a species' genome is shown as a contiguous run of probes with green hybridization intensities, red indicates an amplified region, and black indicates a balanced complement of both parental species' genomes. The arrows a–d indicate the following deletion events: a) a deletion on *S. cerevisiae* chromosome V between two Ty1 elements; b) a deletion in *S. cerevisiae* chromosome XII between two Ty1 elements; c) a deletion at the left end of the *S. cerevisiae* chromosome XV; d) a deletion at the right end of the *S. uvarum* chromosome IX, that encompasses the *DAL3* locus. The solid black bars indicate the regions on chromosome XIV that underwent the recurrent non-reciprocal translocation events that resulted in the *MEP2* fusion gene.
(PDF)

Figure S3 Alignment of *MEP2* gene and protein sequences for ancestral and evolved clones. (A) Alignment of *MEP2* gene sequences for *S. cerevisiae*, *S. uvarum*, and three evolved clones containing *MEP2* fusion genes. The *MEP2* gene DNA sequences for the indicated strains (in all cases the same length) were aligned by MUSCLE [98], with sequences for *S. uvarum* shown in blue font and for *S. cerevisiae* in red. Identical nucleotides at a given position that are shared between all the sequences are denoted with an asterisk below the sequence. The ATG and stop codon (TAA) are shown in bold red font. For each evolved clone, the region of the gene in which the fusion junction occurred is shown in bold green underlined font plus a yellow-highlighted run of asterisks below; the actual fusion junction had to occur somewhere within the highlighted region, but it is impossible to determine the exact nucleotide since the region is identical between the two species. (B) Alignment of Mep2 protein sequences for *S. cerevisiae*, *S. uvarum*, and GSY2532, 2533, and 2535. The Mep2 protein sequences for the indicated strains (in all cases proteins have the same lengths) were aligned using MUSCLE and are shown in the same order as for Figure S3A. Below the sequence, amino acids identical between all strains are shown as an asterisk, conservative amino acid differences are shown as a colon, semi-conservative as a period, and non-conservative as a blank. To aid in visualization of protein sequence differences between the two parent species and between the evolved fusion proteins, amino acids that vary between the two species are highlighted, with the amino acid corresponding to the *S. uvarum* protein sequence in turquoise and *S. cerevisiae* in yellow.
(PDF)

Figure S4 Many recurrent independent rearrangements of the *MEP2* locus during evolution. (A) Distribution of types of PCR products from many population clones. From each of Vessels A and B, twelve single-colony clones per time point (0, ∼50, ∼100, ∼150 and ∼200 generations) were isolated and tested by PCR for the presence or absence of 4 different products, using primers specific to each species located at the 3′ and 5′ ends of the *MEP2* gene (listed in Table S1 as “genotyping” primers). The 4 different products were: *S. cerevisiae MEP2* gene, *S. uvarum MEP2* gene, *S. cerevisiae* - *S. uvarum* fusion *MEP2* gene, and the *S. uvarum* - *S. cerevisiae* “reverse” fusion *MEP2* gene. The *S. uvarum* - *S. cerevisiae* “reverse” fusion *MEP2* gene was never observed. Results for Vessel A clones are shown above and for Vessel B below. For each time-point the proportion of clones containing various combinations of *MEP2* genes is shown, with gene combinations colored according to the legend. “Ancestral” (green) = *S. cerevisiae MEP2* gene plus *S. uvarum MEP2* gene; “Fusion Only” (blue) = presence of only the *S. cerevisiae* - *S. uvarum* fusion *MEP2* gene; “Fusion+*S. uvarum*” (yellow) = presence of the *S. cerevisiae* - *S. uvarum* fusion *MEP2* gene plus *S. uvarum MEP2* gene; “*S. uvarum* only” (orange) = presence of *S. uvarum MEP2* gene only; “*S. cerevisiae* only” (red) = presence of *S. cerevisiae MEP2* gene only. Note that we never observed the “Fusion+*S. cerevisiae*” combination. (B) Fusion junctions from PCR product sequencing of many population clones. The *MEP2* gene sequences for *S. uvarum* (in blue font) and for *S. cerevisiae* (in orange font) are shown along the top of each line, with asterisks for shared nucleotides shown below in three rows, indicating Vessels A, B or C. We obtained junction sequences by Sanger sequencing *MEP2* fusion-gene PCR products from multiple clones from various time points: 17 clones from Vessel A and 18 from Vessel B (note: aside from GSY2535, we did not investigate any further Vessel C clones). In the figure, we indicate the fusion junctions we found by yellow highlighting of the asterisks (for the vessel(s) in which it occurred) corresponding to the junction region. One fusion junction was found in common between Vessels A and B (highlighted for both rows). Fusion junctions for clones GSY2532, GSY2533, and GSY2535 are also shown. Under each highlighted fusion junction is shown the number of clones for which that junction was observed (and the population generation time point from which the clone(s) were derived), except for three junctions where there are too many to report; those results given here: “GSY2532 junction”: 1 clone from ∼100 generations, 1 clone from ∼150 generations, 4 clones from ∼200 generations (including GSY2532). “GSY2533 junction”: 7 clones from ∼150 generations, 5 clones from ∼200 generations (including GSY2533). Junction found in both Vessels A and B: Vessel A: 1 clone from ∼100 generations, 6 clones from ∼150 generations, 1 clone from ∼200 generations. Vessel B: 3 clones from ∼100 generations.
(PDF)

Figure S5 Histograms of qRT-PCR results. (A) Histogram of averaged expression levels of unevolved and evolved *MEP2* genes. Depicted are the fold-expressions (shown as averages of biological and technical replicates; see below) of *S. cerevisiae MEP2* and *S. uvarum MEP2* genes from the unevolved *S. cerevisiae* - *S. uvarum* interspecific hybrid GSY86, as well as the fold-expression of the *MEP2* fusion gene from an evolved hybrid (GSY2532). qRT-PCR was performed on RNA isolated from yeast growing at steady state under ammonium limitation. Before averaging, all data were normalized relative to an *S. uvarum* control gene measured in both strains, and then normalized to the *S. uvarum MEP2* gene from the *S. cerevisiae* - *S. uvarum* interspecific hybrid GSY86 (Δ Δ Ct values). The bar on the far right indicates the “per-locus” expression for the *MEP2* fusion gene in the evolved hybrid (GSY2532), obtained by dividing the measured expression levels by two. This adjustment is not required for the *S. cerevisiae MEP2* and *S. uvarum MEP2* genes from the *S. cerevisiae* - *S. uvarum* interspecific hybrid, GSY86, because they exist as uniquely-monitored single loci and are thus by definition already measured at a “per-locus” expression level. (B) Data for individual biological replicates. Expression levels for individual biological replicates (2 to 3 technical replicates per biological replicate) are shown; these data were averaged to produce the histogram in (A). See Table S4 for the full dataset.
(PDF)

Figure S6 Additional recombination models that lead to evolved clones' genomic configuration. (A) Alternative recombination model involving two BIR events. Labeling and abbreviations are as in Figure 4. This two-step recombination model involves two successive BIR events. In the first event, a break in the coding region of *MEP2* in the S. *uvarum* chromosome is repaired using the *S. cerevisiae* chromosome as a template, resulting in the left end of chromosome 14 being replaced. A second BIR event, this time in the *S. cerevisiae* chromosome, but downstream of *MEP2*, is then repaired using the existing fusion chromosome as a template, yielding 2 *MEP2* fusion genes with identical fusion points. We consider this model unlikely as it requires an intermediate stage that contains both a *MEP2* fusion gene and an intact *S. cerevisiae MEP2* gene, which we never observed. (B) Alternative non-BIR recombination models. Labeling and abbreviations are as in Figure 4, with the addition that “RevFusion” refers to the “reverse fusion” *MEP2* as described below. This two-step recombination model involves two successive mitotic crossover (“CO”) events. The first step involves a crossover between a *S. cerevisiae* and a *S. uvarum* chromosome in a G2 cell, occurring within the coding sequence of the *MEP2* gene. This is followed by segregation of two alternative sets of chromatids, shown by the thin red arrows pointing to either the left or right sides. The left hand panels show one possible route to the final evolved clone configuration, which starts with a cell that contains a *MEP2* fusion gene and an intact *S. cerevisiae MEP2* gene, which undergoes a subsequent crossover between the chimaeric chromosome and an intact *S. cerevisiae* chromosome in a G2 cell, with segregation of the red arrow chromatids resulting in a cell with the final evolved state of two copies of the *MEP2* fusion gene and no copies of either parental *MEP2* gene. The right hand path is similar, with a second G2 mitotic crossover event and segregation leading to the final configuration, except that the starting cell contains a *S. cerevisiae* - *S. uvarum MEP2* fusion gene as well as a “reverse” fusion, i.e., a *S. uvarum* - *S. cerevisiae MEP2* fusion gene. We believe the models depicted in this figure are unlikely because we never observed clones from any of the evolution time points that contained both a *S. cerevisiae* - *S. uvarum MEP2* fusion gene and an intact *S. cerevisiae MEP2* gene, or that ever contained a “reverse” fusion *S. uvarum* - *S. cerevisiae MEP2* gene.
(PDF)

Table S1 Primers used in this study. At the top are listed the primers used in various combinations for “genotyping”, i.e., distinguishing the *S. cerevisiae MEP2* gene, *S. uvarum MEP2* gene, *S. cerevisiae-S. uvarum* fusion *MEP2* gene, and *S. uvarum*-*S. cerevisiae* “reverse fusion” *MEP2* gene. Primers marked as “sequencing” were used for targeted Sanger sequencing of the PCR products that were determined to arise from a *S. cerevisiae-S. uvarum* fusion *MEP2* gene. Primers used for Quantitative Reverse-Transcription PCR (qRT-PCR) are shown at the bottom, for the *S. cerevisiae MEP2* gene, *S. uvarum MEP2* gene, and for two control genes, the *S. cerevisiae TFC1* and *S. uvarum* YDR458C genes. GSP number refers to the laboratory primer collection number.
(DOCX)

Table S2 Whole genome sequencing SNP analysis. Results of single nucleotide polymorphism (SNP) detection analysis from whole genome sequencing results for 200-generation evolved clones GSY2532, GSY2533, and GSY2535, performed as described in the text. Determination of conservative, semi-conservative and non-conservative amino acid changes were made according to http://www.clustal.org/download/clustalx_help.html.
(PDF)

Table S3 Physiological phenotypes of the ancestral interspecific hybrid in relation to three independently evolved clones. Estimates of cell density (A_600_), biomass and residual glucose were derived from culturing single colony isolates of the ancestral *S. cerevisiae*×*S. uvarum* interspecific hybrid (GSY86) and 3 evolved isolates (GSY2532, GSY2533, GSY2535). Samples were removed from aerobic, ammonium-limited chemostats at steady state, D = 0.16 h^−1^. We used a one-way ANOVA followed by a Student-Newman-Kuels test to test for significant differences. Different superscript lettering indicates resolved significant differences between isolates; n = 3. For all strains, residual ammonium was near or below the assay detection limit (0.01 ppm).
(PDF)

Table S4 qPCR data for *MEP2* genes. Raw and calculated qPCR data are given for the *S. cerevisiae*, and *S. uvarum MEP2* genes in GSY86, as well as the *MEP2* fusion gene in an evolved clone, GSY2532. To compare across strains, a control *S. uvarum* gene, YDR458C was measured in both strains.
(PDF)
