# Supplementary figures and images for: Recurrent Rearrangement during Adaptive Evolution in an Interspecific Yeast Hybrid Suggests a Model for Rapid Introgression
Source: PLoS Genet. 2013 Mar 21;9(3):e1003366. doi: 10.1371/journal.pgen.1003366 (PMC3605161; doi:10.1371/journal.pgen.1003366)

Supp. Figure 1

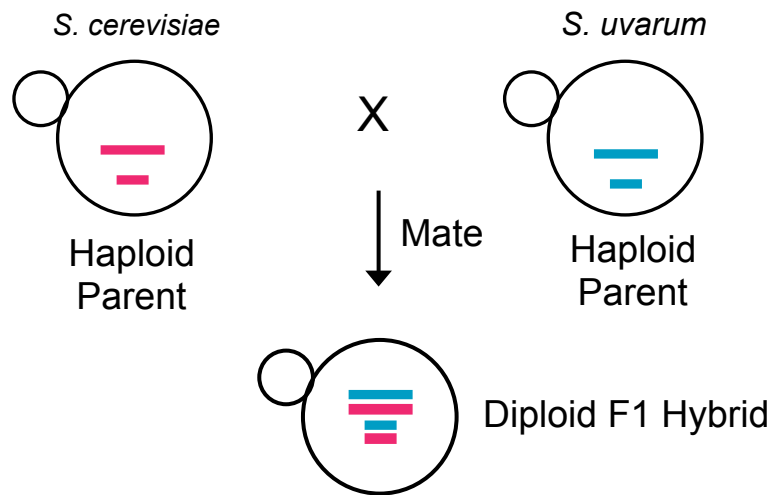

Supplement: Figure S1 — Creating the hybrid strain for experimental evolution. Haploid S. cerevisiae and S. uvarum are mated to produce a diploid F1 hybrid. For simplicity, only 2 chromosomes are shown per cell, instead of the 16 chromosomes normally found in a haploid Saccharomyces cell. (PDF) [file pgen.1003366.s001.pdf]

Figure S2

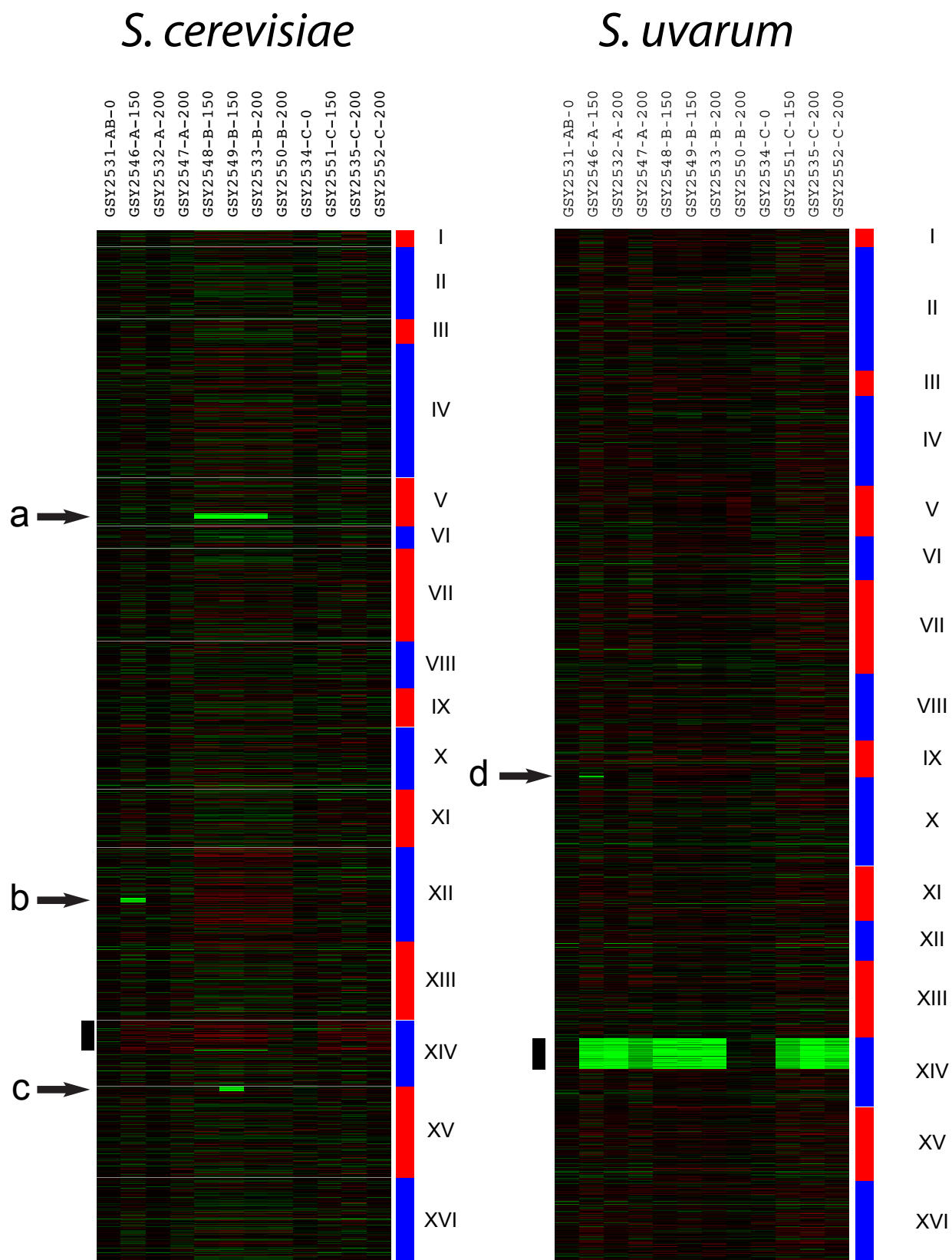

Supplement: Figure S2 — Array-CGH data for F1 founders and evolved clones. Each column contains the aCGH hybridization data for a given strain (details about each strain can be found in Table 1), while each row corresponds to a probe for a chromosomal location; note that the data for each species were normalized separately. Each parental genome is shown separately, labeled at top, with probes ordered downward in chromosomal order from the left end of Chromosome I (top-most probe) to the right end of Chromosome XVI (bottom probe). The deletion of a region of a species' genome is shown as a contiguous run of probes with green hybridization intensities, red indicates an amplified region, and black indicates a balanced complement of both parental species' genomes. The arrows a–d indicate the following deletion events: a) a deletion on S. cerevisiae chromosome V between two Ty1 elements; b) a deletion in S. cerevisiae chromosome XII between two Ty1 elements; c) a deletion at the left end of the S. cerevisiae chromosome XV; d) a deletion at the right end of the S. uvarum chromosome IX, that encompasses the DAL3 locus. The solid black bars indicate the regions on chromosome XIV that underwent the recurrent non-reciprocal translocation events that resulted in the MEP2 fusion gene. (PDF) [file pgen.1003366.s002.pdf]

Figure S5

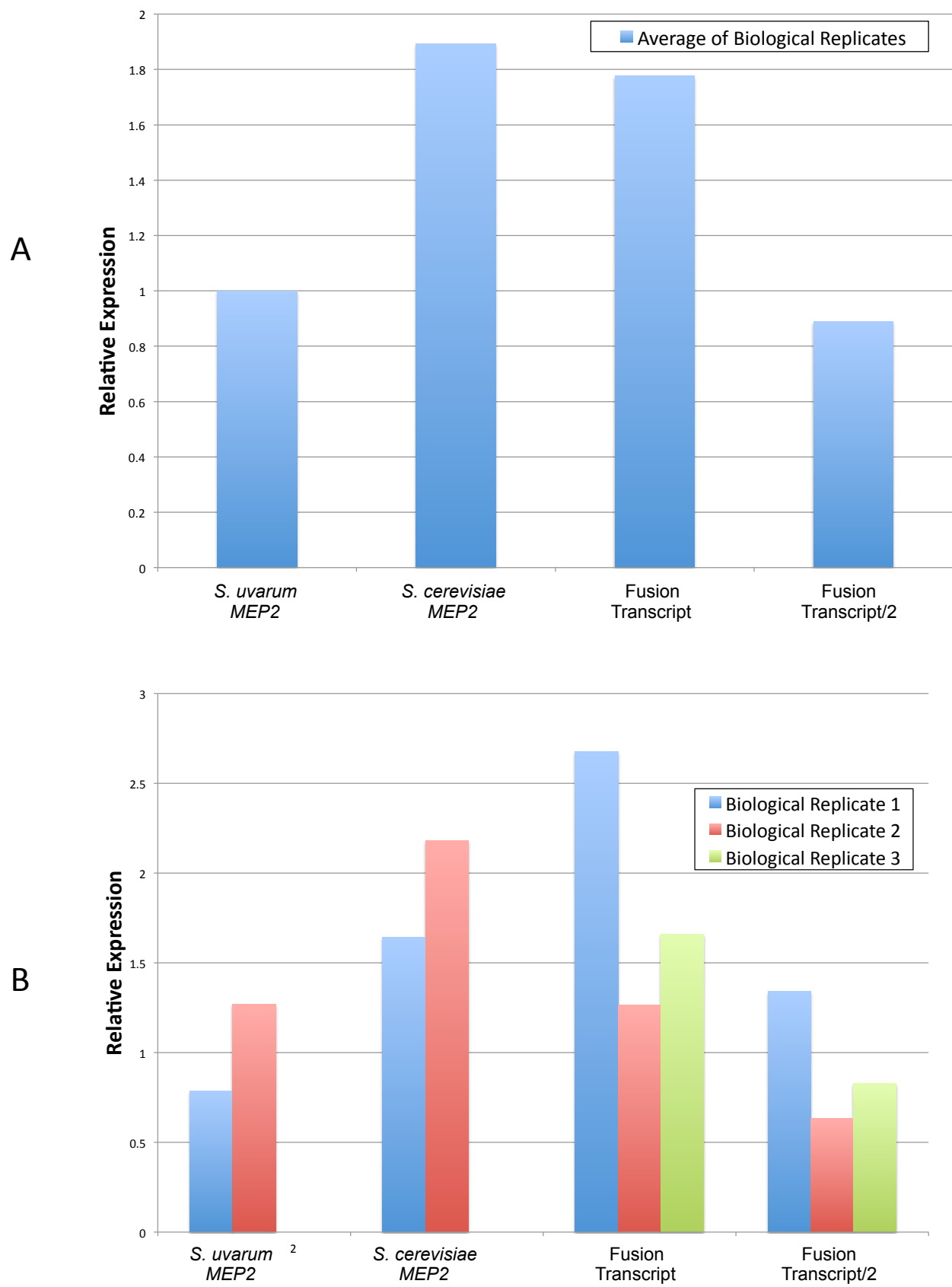

Supplement: Figure S5 — Histograms of qRT-PCR results. (A) Histogram of averaged expression levels of unevolved and evolved MEP2 genes. Depicted are the fold-expressions (shown as averages of biological and technical replicates; see below) of S. cerevisiae MEP2 and S. uvarum MEP2 genes from the unevolved S. cerevisiae - S. uvarum interspecific hybrid GSY86, as well as the fold-expression of the MEP2 fusion gene from an evolved hybrid (GSY2532). qRT-PCR was performed on RNA isolated from yeast growing at steady state under ammonium limitation. Before averaging, all data were normalized relative to an S. uvarum control gene measured in both strains, and then normalized to the S. uvarum MEP2 gene from the S. cerevisiae - S. uvarum interspecific hybrid GSY86 (Δ Δ Ct values). The bar on the far right indicates the “per-locus” expression for the MEP2 fusion gene in the evolved hybrid (GSY2532), obtained by dividing the measured expression levels by two. This adjustment is not required for the S. cerevisiae MEP2 and S. uvarum MEP2 genes from the S. cerevisiae - S. uvarum interspecific hybrid, GSY86, because they exist as uniquely-monitored single loci and are thus by definition already measured at a “per-locus” expression level. (B) Data for individual biological replicates. Expression levels for individual biological replicates (2 to 3 technical replicates per biological replicate) are shown; these data were averaged to produce the histogram in (A). See Table S4 for the full dataset. (PDF) [file pgen.1003366.s005.pdf]

Supp. Figure 6A

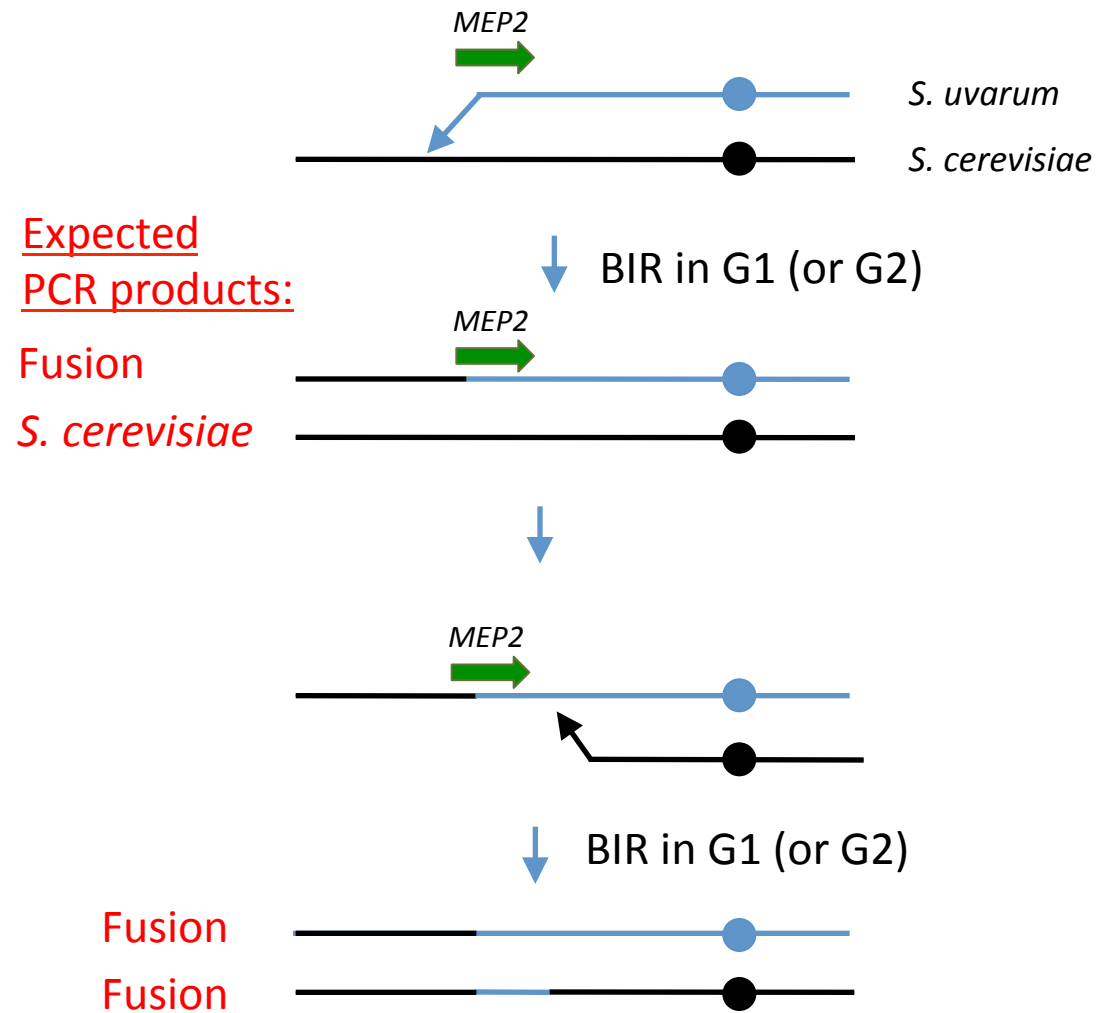

Supp. Figure 6B

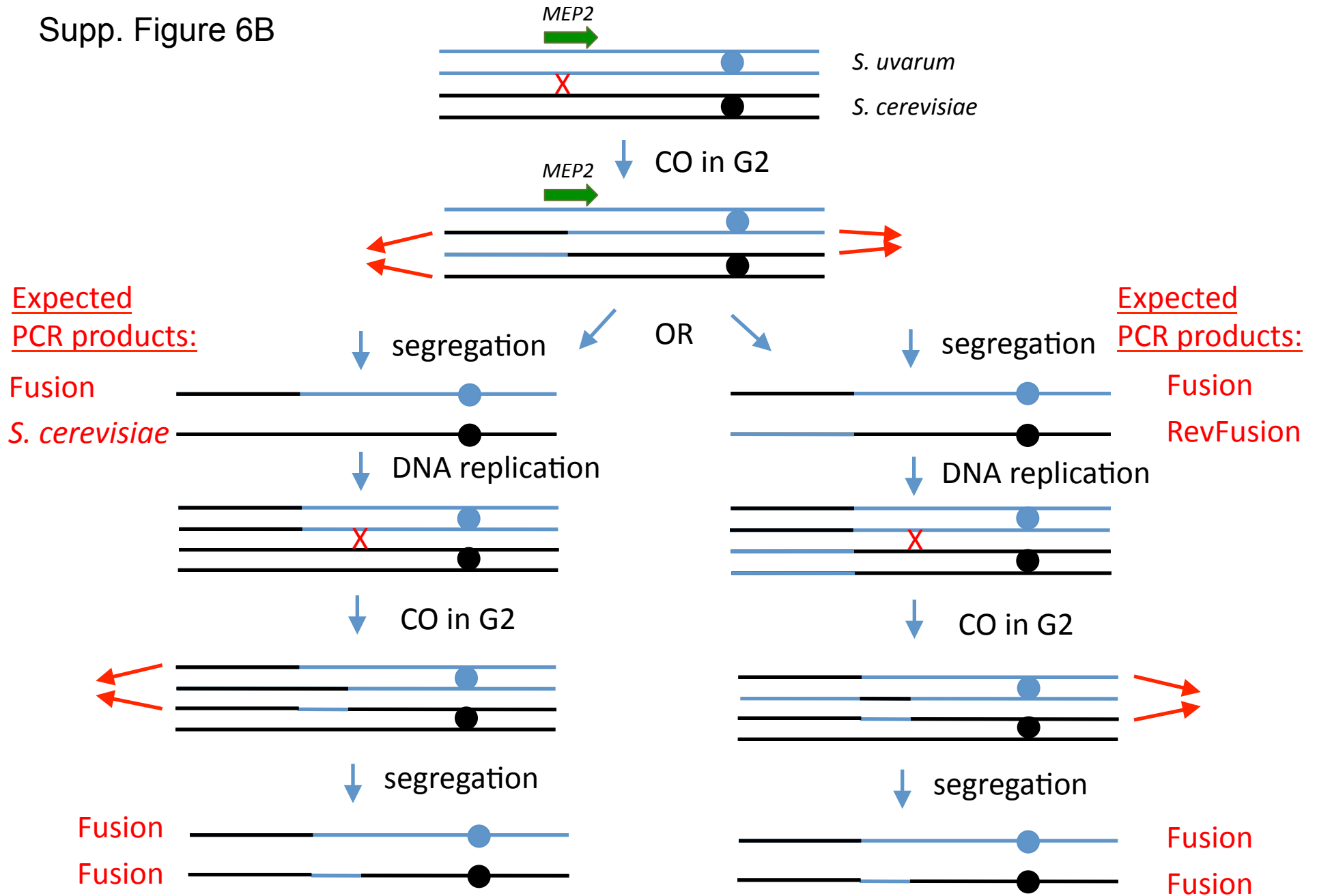

Supplement: Figure S6 — Additional recombination models that lead to evolved clones' genomic configuration. (A) Alternative recombination model involving two BIR events. Labeling and abbreviations are as in Figure 4. This two-step recombination model involves two successive BIR events. In the first event, a break in the coding region of MEP2 in the S. uvarum chromosome is repaired using the S. cerevisiae chromosome as a template, resulting in the left end of chromosome 14 being replaced. A second BIR event, this time in the S. cerevisiae chromosome, but downstream of MEP2, is then repaired using the existing fusion chromosome as a template, yielding 2 MEP2 fusion genes with identical fusion points. We consider this model unlikely as it requires an intermediate stage that contains both a MEP2 fusion gene and an intact S. cerevisiae MEP2 gene, which we never observed. (B) Alternative non-BIR recombination models. Labeling and abbreviations are as in Figure 4, with the addition that “RevFusion” refers to the “reverse fusion” MEP2 as described below. This two-step recombination model involves two successive mitotic crossover (“CO”) events. The first step involves a crossover between a S. cerevisiae and a S. uvarum chromosome in a G2 cell, occurring within the coding sequence of the MEP2 gene. This is followed by segregation of two alternative sets of chromatids, shown by the thin red arrows pointing to either the left or right sides. The left hand panels show one possible route to the final evolved clone configuration, which starts with a cell that contains a MEP2 fusion gene and an intact S. cerevisiae MEP2 gene, which undergoes a subsequent crossover between the chimaeric chromosome and an intact S. cerevisiae chromosome in a G2 cell, with segregation of the red arrow chromatids resulting in a cell with the final evolved state of two copies of the MEP2 fusion gene and no copies of either parental MEP2 gene. The right hand path is similar, with a second G2 mitotic crossover event and segregat [file pgen.1003366.s006.pdf]
