# Supplementary material for: Recurrent Rearrangement during Adaptive Evolution in an Interspecific Yeast Hybrid Suggests a Model for Rapid Introgression
Source: PLoS Genet. 2013 Mar 21;9(3):e1003366. doi: 10.1371/journal.pgen.1003366 (PMC3605161; doi:10.1371/journal.pgen.1003366)
Supplement: Figure S3 — Alignment of MEP2 gene and protein sequences for ancestral and evolved clones. (A) Alignment of MEP2 gene sequences for S. cerevisiae, S. uvarum, and three evolved clones containing MEP2 fusion genes. The MEP2 gene DNA sequences for the indicated strains (in all cases the same length) were aligned by MUSCLE [98], with sequences for S. uvarum shown in blue font and for S. cerevisiae in red. Identical nucleotides at a given position that are shared between all the sequences are denoted with an asterisk below the sequence. The ATG and stop codon (TAA) are shown in bold red font. For each evolved clone, the region of the gene in which the fusion junction occurred is shown in bold green underlined font plus a yellow-highlighted run of asterisks below; the actual fusion junction had to occur somewhere within the highlighted region, but it is impossible to determine the exact nucleotide since the region is identical between the two species. (B) Alignment of Mep2 protein sequences for S. cerevisiae, S. uvarum, and GSY2532, 2533, and 2535. The Mep2 protein sequences for the indicated strains (in all cases proteins have the same lengths) were aligned using MUSCLE and are shown in the same order as for Figure S3A. Below the sequence, amino acids identical between all strains are shown as an asterisk, conservative amino acid differences are shown as a colon, semi-conservative as a period, and non-conservative as a blank. To aid in visualization of protein sequence differences between the two parent species and between the evolved fusion proteins, amino acids that vary between the two species are highlighted, with the amino acid corresponding to the S. uvarum protein sequence in turquoise and S. cerevisiae in yellow. (PDF) [file pgen.1003366.s003.pdf]

Supp. Figure 3A

```
S. uvarum             CAACATAAACAATGTCTTACAATTTTACAGGTACGCCCACGGGCGAAGGAACGGGTGGTA   60
S. cerevisiae         CAATATCAACAATGTCTTACAATTTTACAGGTACGCCTACAGGCGAAGGAACGGGTGGTA   60
GSY2532 (Vessel A)    CAATATCAACAATGTCTTACAATTTTACAGGTACGCCTACAGGCGAAGGAACGGGTGGTA   60
GSY2533 (Vessel B)    CAATATCAACAATGTCTTACAATTTTACAGGTACGCCTACAGGCGAAGGAACGGGTGGTA   60
GSY2535 (Vessel C)    CAATATCAACAATGTCTTACAATTTTACAGGTACGCCTACAGGCGAAGGAACGGGTGGTA   60
                      *** ** ****************************** ** *******************   60

S. uvarum             ACTCATTGACCACAGACTTAAACACCCAATATGATTTGGCTAATATGGGATGGATCGGTG  120
S. cerevisiae         ACTCGTTGACAACAGATTTGAATACACAATTTGACTTGGCCAACATGGGATGGATCGGTG  120
GSY2532               ACTCGTTGACAACAGATTTGAATACACAATTTGACTTGGCCAACATGGGATGGATCGGTG  120
GSY2533               ACTCGTTGACAACAGATTTGAATACACAATTTGACTTGGCCAACATGGGATGGATCGGTG  120
GSY2535               ACTCGTTGACAACAGATTTGAATACACAATTTGACTTGGCCAACATGGGATGGATCGGTG  120
                      **** ***** ***** ** ** ** **** *** ***** ** ****************  120

S. uvarum             TAGCTTCCGCAGGTGTCTGGATTATGGTGCCAGGTATCGGTCTGTTATACTCCGGTTTGT  180
S. cerevisiae         TGGCTTCAGCAGGTGTGTGGATTATGGTCCCAGGTATCGGTTTATTATATTCTGGTTTAT  180
GSY2532               TGGCTTCAGCAGGTGTGTGGATTATGGTCCCAGGTATCGGTTTATTATATTCTGGTTTAT  180
GSY2533               TGGCTTCAGCAGGTGTGTGGATTATGGTCCCAGGTATCGGTTTATTATATTCTGGTTTAT  180
GSY2535               TGGCTTCAGCAGGTGTGTGGATTATGGTCCCAGGTATCGGTTTATTATATTCTGGTTTAT  180
                      * ***** ******** *********** ************ * ***** ** ***** *  180

S. uvarum             CTAGGAAAAAGCACGCTTTATCTTTGCTTTGGGCCTCGATGATGGCCTCTGCAGTGTGTA  240
S. cerevisiae         CCAGGAAAAAGCATGCTTTATCCTTGCTTTGGGCCTCGATGATGGCTTCCGCCGTGTGTA  240
GSY2532               CCAGGAAAAAGCATGCTTTATCCTTGCTTTGGGCCTCGATGATGGCTTCCGCCGTGTGTA  240
GSY2533               CCAGGAAAAAGCATGCTTTATCCTTGCTTTGGGCCTCGATGATGGCTTCCGCCGTGTGTA  240
GSY2535               CCAGGAAAAAGCATGCTTTATCCTTGCTTTGGGCCTCGATGATGGCTTCCGCCGTGTGTA  240
                      * *********** ******** *********************** ** ** *******  240

S. uvarum             TCTTCCAGTGGTTTTTCTGGGGGTATTCGCTGGCCTTCTCACATAACACTAGAGGTCATG  300
S. cerevisiae         TTTTCCAATGGTTTTTCTGGGGATACTCATTAGCTTTCTCACACAACACTAGAGGTAACG  300
GSY2533               TTTTCCAATGGTTTTTCTGGGGATACTCATTAGCTTTCTCACACAACACTAGAGGTAACG  300
GSY2532               TTTTCCAATGGTTTTTCTGGGGATACTCATTAGCTTTCTCACACAACACTAGAGGTAACG  300
GSY2535               TTTTCCAATGGTTTTTCTGGGGATACTCATTAGCTTTCTCACACAACACTAGAGGTAACG  300
                      * ***** ************** ** **  * ** ******** ************ * *  300

S. uvarum             GTTTCATTGGTACTTTGGAATTCTTCGGGTTCCGTAACGTTTTGGGGGCTCCCTCCAGTG  360
S. cerevisiae         GTTTTATTGGTACCTTGGAATTCTTTGGGTTTCGTAACGTTTTAGGAGCCCCATCTAGTG  360
GSY2532               GTTTTATTGGTACCTTGGAATTCTTTGGGTTTCGTAACGTTTTAGGAGCCCCATCTAGTG  360
GSY2533               GTTTTATTGGTACCTTGGAATTCTTTGGGTTTCGTAACGTTTTAGGAGCCCCATCTAGTG  360
GSY2535               GTTTTATTGGTACCTTGGAATTCTTTGGGTTTCGTAACGTTTTAGGAGCCCCATCTAGTG  360
                      **** ******** *********** ***** *********** ** ** ** ** ****  360

S. uvarum             TCAGCTCATTGCCTGACATCTTATTTGCTGTTTACCAAGGTATGTTTGCCGCCGTCACCG  420
S. cerevisiae         TCAGTTCTCTTCCCGATATACTGTTTGCCGTTTACCAAGGTATGTTTGCCGCAGTCACCG  420
GSY2532               TCAGTTCTCTTCCCGATATACTGTTTGCCGTTTACCAAGGTATGTTTGCCGCAGTCACCG  420
GSY2533               TCAGTTCTCTTCCCGATATACTGTTTGCCGTTTACCAAGGTATGTTTGCCGCAGTCACCG  420
GSY2535               TCAGTTCTCTTCCCGATATACTGTTTGCCGTTTACCAAGGTATGTTTGCCGCAGTCACCG  420
                      **** **  * ** ** **  * ***** *********************** *******  420

S. uvarum             GTGCCCTAATGTTGGGTGGTGCTTGCGAAAGAGCAAGACTGTTCCCCATGATGGTGTTCT  480
S. cerevisiae         GTGCCCTAATGCTAGGTGGTGCCTGCGAGAGGGCAAGGTTGTTTCCTATGATGGTGTTCT  480
GSY2532               GTGCCCTAATGCTAGGTGGTGCCTGCGAGAGGGCAAGGTTGTTTCCTATGATGGTGTTCT  480
GSY2533               GTGCCCTAATGCTAGGTGGTGCCTGCGAGAGGGCAAGGTTGTTTCCTATGATGGTGTTCT  480
GSY2535               GTGCCCTAATGCTAGGTGGTGCCTGCGAGAGGGCAAGGTTGTTTCCTATGATGGTGTTCT  480
                      *********** * ******** ***** ** *****  **** ** *************  480
```

Supp. Figure 3A

```
S. uvarum       TATTCTTATGGATGACTGTCGTCTACTGTCCAATTGCTTGCTGGGTCTGGAACGCAGAAG  540
S. cerevisiae   TGTTTTTATGGATGACTATTGTTTATTGTCCTATTGCATGCTGGGTCTGGAATGCCGAGG  540
GSY2532         TGTTTTTATGGATGACTGTCGTCTACTGTCCAATTGCTTGCTGGGTCTGGAACGCAGAAG  540
GSY2533         TGTTTTTATGGATGACTATTGTTTATTGTCCTATTGCATGCTGGGTCTGGAATGCCGAGG  540
GSY2535         TGTTTTTATGGATGACTATTGTTTATTGTCCTATTGCATGCTGGGTCTGGAATGCCGAGG  540
                * ** ************ * ** ** ***** ***** ************** ** ** *  540

S. uvarum       GCTGGTTGGTCAAGTTAGGCAGTTTGGACTATGCTGGTGGGTTATGTGTTCATTTGACTT  600
S. cerevisiae   GTTGGTTGGTCAAATTGGGTAGCTTGGACTATGCAGGTGGTTTATGTGTCCATTTAACAT  600
GSY2532         GCTGGTTGGTCAAGTTAGGCAGTTTGGACTATGCTGGTGGGTTATGTGTTCATTTGACTT  600
GSY2533         GTTGGTTGGTCAAATTGGGTAGCTTGGACTATGCAGGTGGTTTATGTGTCCATTTAACAT  600
GSY2535         GTTGGTTGGTCAAATTGGGTAGCTTGGACTATGCTGGTGGGTTATGTGTTCATTTGACTT  600
                * *********** ** ** ** *********** ***** ******** ***** ** *  600

S. uvarum       CCGGCCATGGTGGGTTGGTCTACGCCTTGATATTGGGTAGACGTAACGACCCTGTGACAC  660
S. cerevisiae   CTGGACATGGTGGTCTAGTTTACGCTTTGATACTGGGTAAGCGTAATGACCCTGTTACAC  660
GSY2532         CCGGCCATGGTGGGTTGGTCTACGCCTTGATATTGGGTAGACGTAACGACCCTGTGACAC  660
GSY2533         CTGGACATGGTGGTCTAGTTTACGCTTTGATACTGGGTAAGCGTAATGACCCTGTTACAC  660
GSY2535         CCGGCCATGGTGGGTTGGTCTACGCCTTGATATTGGGTAGACGTAACGACCCTGTGACAC  660
                * ** ********  * ** ***** ****** ******  ***** ******** ****  660

S. uvarum       GCAAGGGAATGCCCAAGTACAAGCCACATTCCGTCACCTCCGTGGTGTTAGGAACTGTGT  720
S. cerevisiae   GTAAAGGGATGCCCAAGTACAAACCACATTCCGTCACCTCGGTGGTTTAGGCACAGTGT   720
GSY2532         GCAAGGGAATGCCCAAGTACAAGCCACATTCCGTCACCTCCGTGGTGTTAGGAACTGTGT  720
GSY2533         GTAAAGGGATGCCCAAGTACAAACCACATTCCGTCACCTCGGTGGTTTAGGCACAGTGT   720
GSY2535         GCAAGGGAATGCCCAAGTACAAGCCACATTCCGTCACCTCCGTGGTGTTAGGAACTGTGT  720
                * ** ** ************** ***************** ***** ***** ** ****  720

S. uvarum       TTTTATGGTTCGGTTGGATGTTCTTCAACGGTGGCTCTGCCGGGAATGCCACCATACGAG  780
S. cerevisiae   TCTTATGGTTTGGTTGGATGTTCTTTAACGGAGGCTCTGCAGGTAATGCAACTATACGAG  780
GSY2532         TTTTATGGTTCGGTTGGATGTTCTTCAACGGTGGCTCTGCCGGGAATGCCACCATACGAG  780
GSY2533         TCTTATGGTTTGGTTGGATGTTCTTTAACGGAGGCTCTGCAGGTAATGCAACTATACGAG  780
GSY2535         TTTTATGGTTCGGTTGGATGTTCTTCAACGGTGGCTCTGCCGGGAATGCCACCATACGAG  780
                * ******** ************** ***** ******** ** ***** ** *******  780

S. uvarum       CATGGTACTCTATAATGTCCACCAACTTGGCTGCTGCTTGCGGTGGGTTGACCTGGATGG  840
S. cerevisiae   CATGGTACTCTATTATGTCCACAAACTTAGCTGCTGCTTGCGGTGGCTTGACATGGATGG  840
GSY2532         CATGGTACTCTATAATGTCCACCAACTTGGCTGCTGCTTGCGGTGGGTTGACCTGGATGG  840
GSY2533         CATGGTACTCTATTATGTCCACAAACTTAGCTGCTGCTTGCGGTGGCTTGACATGGATGG  840
GSY2535         CATGGTACTCTATAATGTCCACCAACTTGGCTGCTGCTTGCGGTGGGTTGACCTGGATGG  840
                ************* ******** ***** ***************** ***** *******  840

S. uvarum       TGATCGATTATTTCAGATCTGGTAGAAAGTGGACCACTGTCGGTCTATGTTCAGGTATCA  900
S. cerevisiae   TTATCGATTATTTCAGATGCGGAAGAAAGTGGACTACAGTTGGTTTGTGTTCAGGTATCA  900
GSY2532         TGATCGATTATTTCAGATCTGGTAGAAAGTGGACCACTGTCGGTCTATGTTCAGGTATCA  900
GSY2533         TTATCGATTATTTCAGATGCGGAAGAAAGTGGACTACAGTTGGTTTGTGTTCAGGTATCA  900
GSY2535         TGATCGATTATTTCAGATCTGGTAGAAAGTGGACCACTGTCGGTCTATGTTCAGGTATCA  900
                * ****************  ** *********** ** ** *** * *************  900

S. uvarum       TTGCCGGTCTAGTCGGTATCACTCCAGCCGCTGGGTTTGTACCAATCTGGTCTGCCGTTG  960
S. cerevisiae   TCGCTGGCCTAGTGGGTATCACCCCAGCCGCCGGGTTCGTGCCAATCTGGTCAGCCGTTG  960
GSY2532         TTGCCGGTCTAGTCGGTATCACTCCAGCCGCTGGGTTTGTACCAATCTGGTCTGCCGTTG  960
GSY2533         TCGCTGGCCTAGTGGGTATCACCCCAGCCGCCGGGTTCGTGCCAATCTGGTCAGCCGTTG  960
GSY2535         TTGCCGGTCTAGTCGGTATCACTCCAGCCGCTGGGTTTGTACCAATCTGGTCTGCCGTTG  960
                * ** ** ***** ******** ******** ***** ** *********** *******  960
```

Supp. Figure 3A

```
S. uvarum       TCATTGGTGTGGTCACTGGTGCAGGTTGTAATCTTGCTGTTGACTTGAAGGGCTTATTAG 1020
S. cerevisiae   TCATTGGTGTGGTTACTGGTGCAGGATGTAACCTTGCTGTTGACTTAAAGAGTCTATTGC 1020
GSY2532         TCATTGGTGTGGTCACTGGTGCAGGTTGTAATCTTGCTGTTGACTTGAAGGGCTTATTAG 1020
GSY2533         TCATTGGTGTGGTTACTGGTGCAGGATGTAACCTTGCTGTTGACTTAAAGAGTCTATTGC 1020
GSY2535         TCATTGGTGTGGTCACTGGTGCAGGTTGTAATCTTGCTGTTGACTTGAAGGGCTTATTAG 1020
                ************* *********** ***** ************** *** *  ****   1020

S. uvarum       GTATTGACGATGGTTTAGACTGTTACTCTATCCATGGTGTGGGTGCTTGTATTGGCTGTG 1080
S. cerevisiae   GCATCGATGATGGTCTAGATTGTTACTCTATCCATGGTGTGGGTGGTTGTATTGGTTCTG 1080
GSY2532         GTATTGACGATGGTTTAGACTGTTACTCTATCCATGGTGTGGGTGCTTGTATTGGCTGTG 1080
GSY2533         GCATCGATGATGGTCTAGATTGTTACTCTATCCATGGTGTGGGTGCTTGTATTGGCTGTG 1080
GSY2535         GTATTGACGATGGTTTAGACTGTTACTCTATCCATGGTGTGGGTGCTTGTATTGGCTGTG 1080
                * ** ** ****** **** ************************* ********* * ** 1080

S. uvarum       TGCTAACTGGTATCTTTGCCGCTGATTACGTCAATGCCACTGCCGGTTCCTACATCAGTC 1140
S. cerevisiae   TATTAACTGGTATCTTTGCTGCAGACTATGTAAATGCCACTGCAGGCTCTTACATTAGTC 1140
GSY2532         TGCTAACTGGTATCTTTGCCGCTGATTACGTCAATGCCACTGCCGGTTCCTACATCAGTC 1140
GSY2533         TGCTAACTGGTATCTTTGCCGCTGATTACGTCAATGCCACTGCCGGTTCCTACATCAGTC 1140
GSY2535         TGCTAACTGGTATCTTTGCCGCTGATTACGTCAATGCCACTGCCGGTTCCTACATCAGTC 1140
                *  **************** ** ** ** ** *********** ** ** ***** **** 1140

S. uvarum       CCATCGCCGGTGGCTGGATCAACAAACACTACAAACAAGTCGGTTATCAATTGGCTGGTA 1200
S. cerevisiae   CAATTGATGGTGGCTGGATCAATCATCACTATAAACAAGTTGGTTATCAATTAGCAGGTA 1200
GSY2532         CCATCGCCGGTGGCTGGATCAACAAACACTACAAACAAGTCGGTTATCAATTGGCTGGTA 1200
GSY2533         CCATCGCCGGTGGCTGGATCAACAAACACTACAAACAAGTCGGTTATCAATTGGCTGGTA 1200
GSY2535         CCATCGCCGGTGGCTGGATCAACAAACACTACAAACAAGTCGGTTATCAATTGGCTGGTA 1200
                * ** *  **************  * ***** ******** *********** ** **** 1200

S. uvarum       TGTGTGCTGCGCTAGCATGGACCGTTACTGTCACATCTATATTGCTAGTGACTATGAACG 1260
S. cerevisiae   TATGCGCTGCACTAGCCTGGACTGTTACTGTCACATCTATCTTGCTTCTAACTATGAATG 1260
GSY2532         TGTGTGCTGCGCTAGCATGGACCGTTACTGTCACATCTATATTGCTAGTGACTATGAACG 1260
GSY2533         TGTGTGCTGCGCTAGCATGGACCGTTACTGTCACATCTATATTGCTAGTGACTATGAACG 1260
GSY2535         TGTGTGCTGCGCTAGCATGGACCGTTACTGTCACATCTATATTGCTAGTGACTATGAACG 1260
                * ** ***** ***** ***** ***************** *****  * ******** * 1260

S. uvarum       CCATCCCATTTTTGAAGCTAAGATTAAGCGCCGATGAGGAAGAATTAGGTACTGATGCTG 1320
S. cerevisiae   CCATTCCATTTTTAAAACTAAGATTAAGTGCTGATGAGGAAGAATTAGGTACCGACGCTG 1320
GSY2532         CCATCCCATTTTTGAAGCTAAGATTAAGCGCCGATGAGGAAGAATTAGGTACTGATGCTG 1320
GSY2533         CCATCCCATTTTTGAAGCTAAGATTAAGCGCCGATGAGGAAGAATTAGGTACTGATGCTG 1320
GSY2535         CCATCCCATTTTTGAAGCTAAGATTAAGCGCCGATGAGGAAGAATTAGGTACTGATGCTG 1320
                **** ******** ** *********** ** ******************** ** **** 1320

S. uvarum       CTCAAATCGGTGAATTCACCTACGAGGAATCCACCGCTTATATTCCAGAACCAATCAGAT 1380
S. cerevisiae   CTCAAATTGGTGAATTTACATACGAGGAATCCACTGCTTACATCCCAGAACCAATCAGAT 1380
GSY2532         CTCAAATCGGTGAATTCACCTACGAGGAATCCACCGCTTATATTCCAGAACCAATCAGAT 1380
GSY2533         CTCAAATCGGTGAATTCACCTACGAGGAATCCACCGCTTATATTCCAGAACCAATCAGAT 1380
GSY2535         CTCAAATCGGTGAATTCACCTACGAGGAATCCACCGCTTATATTCCAGAACCAATCAGAT 1380
                ******* ******** ** ************** ***** ** **************** 1380

S. uvarum       CAAGAACTTCTGCTCAAATGCCACCTCCTCATGAAACCATCGATGACAAGATCGTTGCTA 1440
S. cerevisiae   CTAAAACATCGGCACAAATGCCACCTCCTCATGAAAACATTGATGATAAGATTGTGGGTA 1440
GSY2532         CAAGAACTTCTGCTCAAATGCCACCTCCTCATGAAACCATCGATGACAAGATCGTTGCTA 1440
GSY2533         CAAGAACTTCTGCTCAAATGCCACCTCCTCATGAAACCATCGATGACAAGATCGTTGCTA 1440
GSY2535         CAAGAACTTCTGCTCAAATGCCACCTCCTCATGAAACCATCGATGACAAGATCGTTGCTA 1440
                * * *** ** ** ********************** *** ***** ***** ** * ** 1440
```

Supp. Figure 3A

```
S. uvarum      ACACAGACGCAGAAAAGAATTCCACGCCTTCTGATGTTTCTTCTACCAAGAACACTGACC 1500
S. cerevisiae  ACACAGACGCAGAAAAGAATTCTACGCCTTCCGACGCTTCTTCTACTAAGAACACTGACC 1500
GSY2532        ACACAGACGCAGAAAAGAATTCCACGCCTTCTGATGTTTCTTCTACCAAGAACACTGACC 1500
GSY2533        ACACAGACGCAGAAAAGAATTCCACGCCTTCTGATGTTTCTTCTACCAAGAACACTGACC 1500
GSY2535        ACACAGACGCAGAAAAGAATTCCACGCCTTCTGATGTTTCTTCTACCAAGAACACTGACC 1500
               ********************** ******** ** * ********* ************* 1500

S. uvarum      ATATAGTATAATGATATACCCGTACAGACTATGCTCTTATTTTTTACTCATTATGTAATC 1560
S. cerevisiae  ATATAGTATAATGATATACTCAAGCAGACTATGATTTTA-TGTTTAATCTTTATGTAACG 1560
GSY2532        ATATAGTATAATGATATACCCGTACAGACTATGCTCTTATTTTTTACTCATTATGTAATC 1560
GSY2533        ATATAGTATAATGATATACCCGTACAGACTATGCTCTTATTTTTTACTCATTATGTAATC 1560
GSY2535        ATATAGTATAATGATATACCCGTACAGACTATGCTCTTATTTTTTACTCATTATGTAATC 1560
               ******************* *   ********* * *** * **** ** ********   1560
```

Supp. Figure 3B

```
S. uvarum         MSYNFTGTPTGEGTGGNSLTTDLNTQYDLANMGWIGVASAGVWIMVPGIGLLYSGLSRKK  60
S. cerevisiae     MSYNFTGTPTGEGTGGNSLTTDLNTQFDLANMGWIGVASAGVWIMVPGIGLLYSGLSRKK  60
GSY2532           MSYNFTGTPTGEGTGGNSLTTDLNTQFDLANMGWIGVASAGVWIMVPGIGLLYSGLSRKK  60
GSY2533           MSYNFTGTPTGEGTGGNSLTTDLNTQFDLANMGWIGVASAGVWIMVPGIGLLYSGLSRKK  60
GSY2535           MSYNFTGTPTGEGTGGNSLTTDLNTQFDLANMGWIGVASAGVWIMVPGIGLLYSGLSRKK  60
                  **************************:*********************************  60

S. uvarum         HALSLLWASMMASAVCIFQWFFWGYSLAFSHNTRGHGFIGTLEFFGFRNVLGAPSSVSSL 120
S. cerevisiae     HALSLLWASMMASAVCIFQWFFWGYSLAFSHNTRGNGFIGTLEFFGFRNVLGAPSSVSSL 120
GSY2532           HALSLLWASMMASAVCIFQWFFWGYSLAFSHNTRGNGFIGTLEFFGFRNVLGAPSSVSSL 120
GSY2533           HALSLLWASMMASAVCIFQWFFWGYSLAFSHNTRGNGFIGTLEFFGFRNVLGAPSSVSSL 120
GSY2535           HALSLLWASMMASAVCIFQWFFWGYSLAFSHNTRGNGFIGTLEFFGFRNVLGAPSSVSSL 120
                  ***********************************:************************ 120

S. uvarum         PDILFAVYQGMFAAVTGALMLGGACERARLFPMMVFLFLWMTVVYCPIACWVWNAEGWLV 180
S. cerevisiae     PDILFAVYQGMFAAVTGALMLGGACERARLFPMMVFLFLWMTIVYCPIACWVWNAEGWLV 180
GSY2532           PDILFAVYQGMFAAVTGALMLGGACERARLFPMMVFLFLWMTVVYCPIACWVWNAEGWLV 180
GSY2533           PDILFAVYQGMFAAVTGALMLGGACERARLFPMMVFLFLWMTIVYCPIACWVWNAEGWLV 180
GSY2535           PDILFAVYQGMFAAVTGALMLGGACERARLFPMMVFLFLWMTIVYCPIACWVWNAEGWLV 180
                  ******************************************:***************** 180

S. uvarum         KLGSLDYAGGLCVHLTSGHGGLVYALILGRRNDPVTRKGMPKYKPHSVTSVVLGTVFLWF 240
S. cerevisiae     KLGSLDYAGGLCVHLTSGHGGLVYALILGKRNDPVTRKGMPKYKPHSVTSVVLGTVFLWF 240
GSY2532           KLGSLDYAGGLCVHLTSGHGGLVYALILGRRNDPVTRKGMPKYKPHSVTSVVLGTVFLWF 240
GSY2533           KLGSLDYAGGLCVHLTSGHGGLVYALILGKRNDPVTRKGMPKYKPHSVTSVVLGTVFLWF 240
GSY2535           KLGSLDYAGGLCVHLTSGHGGLVYALILGRRNDPVTRKGMPKYKPHSVTSVVLGTVFLWF 240
                  *****************************.****************************** 240

S. uvarum         GWMFFNGGSAGNATIRAWYSIMSTNLAAACGGLTWMVIDYFRSGRKWTTVGLCSGIIAGL 300
S. cerevisiae     GWMFFNGGSAGNATIRAWYSIMSTNLAAACGGLTWMVIDYFRCGRKWTTVGLCSGIIAGL 300
GSY2532           GWMFFNGGSAGNATIRAWYSIMSTNLAAACGGLTWMVIDYFRSGRKWTTVGLCSGIIAGL 300
GSY2533           GWMFFNGGSAGNATIRAWYSIMSTNLAAACGGLTWMVIDYFRCGRKWTTVGLCSGIIAGL 300
GSY2535           GWMFFNGGSAGNATIRAWYSIMSTNLAAACGGLTWMVIDYFRSGRKWTTVGLCSGIIAGL 300
                  ******************************************.***************** 300

S. uvarum         VGITPAAGFVPIWSAVVIGVVTGAGCNLAVDLKGLLGIDDGLDCYSIHGVGACIGCVLTG 360
S. cerevisiae     VGITPAAGFVPIWSAVVIGVVTGAGCNLAVDLKSLLRIDDGLDCYSIHGVGGCIGSVLTG 360
GSY2532           VGITPAAGFVPIWSAVVIGVVTGAGCNLAVDLKGLLGIDDGLDCYSIHGVGACIGCVLTG 360
GSY2533           VGITPAAGFVPIWSAVVIGVVTGAGCNLAVDLKSLLRIDDGLDCYSIHGVGACIGCVLTG 360
GSY2535           VGITPAAGFVPIWSAVVIGVVTGAGCNLAVDLKGLLGIDDGLDCYSIHGVGACIGCVLTG 360
                  *********************************.** **************.***.**** 360

S. uvarum         IFAADYVNATAGSYISPIAGGWINKHYKQVGYQLAGMCAALAWTVTVTSILLVTMNAIPF 420
S. cerevisiae     IFAADYVNATAGSYISPIDGGWINHHYKQVGYQLAGICAALAWTVTVTSILLLTMNAIPF 420
GSY2532           IFAADYVNATAGSYISPIAGGWINKHYKQVGYQLAGMCAALAWTVTVTSILLVTMNAIPF 420
GSY2533           IFAADYVNATAGSYISPIAGGWINKHYKQVGYQLAGMCAALAWTVTVTSILLVTMNAIPF 420
GSY2535           IFAADYVNATAGSYISPIAGGWINKHYKQVGYQLAGMCAALAWTVTVTSILLVTMNAIPF 420
                  ****************** *****:***********:***************:******* 420

S. uvarum         LKLRLSADEEELGTDAAQIGEFTYEESTAYIPEPIRSRTSAQMPPPHETIDDKIVANTDA 480
S. cerevisiae     LKLRLSADEEELGTDAAQIGEFTYEESTAYIPEPIRSKTSAQMPPPHENIDDKIVGNTDA 480
GSY2532           LKLRLSADEEELGTDAAQIGEFTYEESTAYIPEPIRSRTSAQMPPPHETIDDKIVANTDA 480
GSY2533           LKLRLSADEEELGTDAAQIGEFTYEESTAYIPEPIRSRTSAQMPPPHETIDDKIVANTDA 480
GSY2535           LKLRLSADEEELGTDAAQIGEFTYEESTAYIPEPIRSRTSAQMPPPHETIDDKIVANTDA 480
                  *************************************.**********.******.**** 480

S. uvarum         EKNSTPSDVSSTKNTDHIV 499
S. cerevisiae     EKNSTPSDASSTKNTDHIV 499
GSY2532           EKNSTPSDVSSTKNTDHIV 499
GSY2533           EKNSTPSDVSSTKNTDHIV 499
GSY2535           EKNSTPSDVSSTKNTDHIV
                  ********.**********
```
