# Supplementary material for: Recurrent Rearrangement during Adaptive Evolution in an Interspecific Yeast Hybrid Suggests a Model for Rapid Introgression
Source: PLoS Genet. 2013 Mar 21;9(3):e1003366. doi: 10.1371/journal.pgen.1003366 (PMC3605161; doi:10.1371/journal.pgen.1003366)
Supplement: Figure S4 — Many recurrent independent rearrangements of the MEP2 locus during evolution. (A) Distribution of types of PCR products from many population clones. From each of Vessels A and B, twelve single-colony clones per time point (0, ∼50, ∼100, ∼150 and ∼200 generations) were isolated and tested by PCR for the presence or absence of 4 different products, using primers specific to each species located at the 3′ and 5′ ends of the MEP2 gene (listed in Table S1 as “genotyping” primers). The 4 different products were: S. cerevisiae MEP2 gene, S. uvarum MEP2 gene, S. cerevisiae - S. uvarum fusion MEP2 gene, and the S. uvarum - S. cerevisiae “reverse” fusion MEP2 gene. The S. uvarum - S. cerevisiae “reverse” fusion MEP2 gene was never observed. Results for Vessel A clones are shown above and for Vessel B below. For each time-point the proportion of clones containing various combinations of MEP2 genes is shown, with gene combinations colored according to the legend. “Ancestral” (green) = S. cerevisiae MEP2 gene plus S. uvarum MEP2 gene; “Fusion Only” (blue) = presence of only the S. cerevisiae - S. uvarum fusion MEP2 gene; “Fusion+S. uvarum” (yellow) = presence of the S. cerevisiae - S. uvarum fusion MEP2 gene plus S. uvarum MEP2 gene; “S. uvarum only” (orange) = presence of S. uvarum MEP2 gene only; “S. cerevisiae only” (red) = presence of S. cerevisiae MEP2 gene only. Note that we never observed the “Fusion+S. cerevisiae” combination. (B) Fusion junctions from PCR product sequencing of many population clones. The MEP2 gene sequences for S. uvarum (in blue font) and for S. cerevisiae (in orange font) are shown along the top of each line, with asterisks for shared nucleotides shown below in three rows, indicating Vessels A, B or C. We obtained junction sequences by Sanger sequencing MEP2 fusion-gene PCR products from multiple clones from various time points: 17 clones from Vessel A and 18 from Vessel B (note: aside from GSY2535, we did not investigate any further Vessel C clones). [file pgen.1003366.s004.pdf]

Supp. Figure 4A

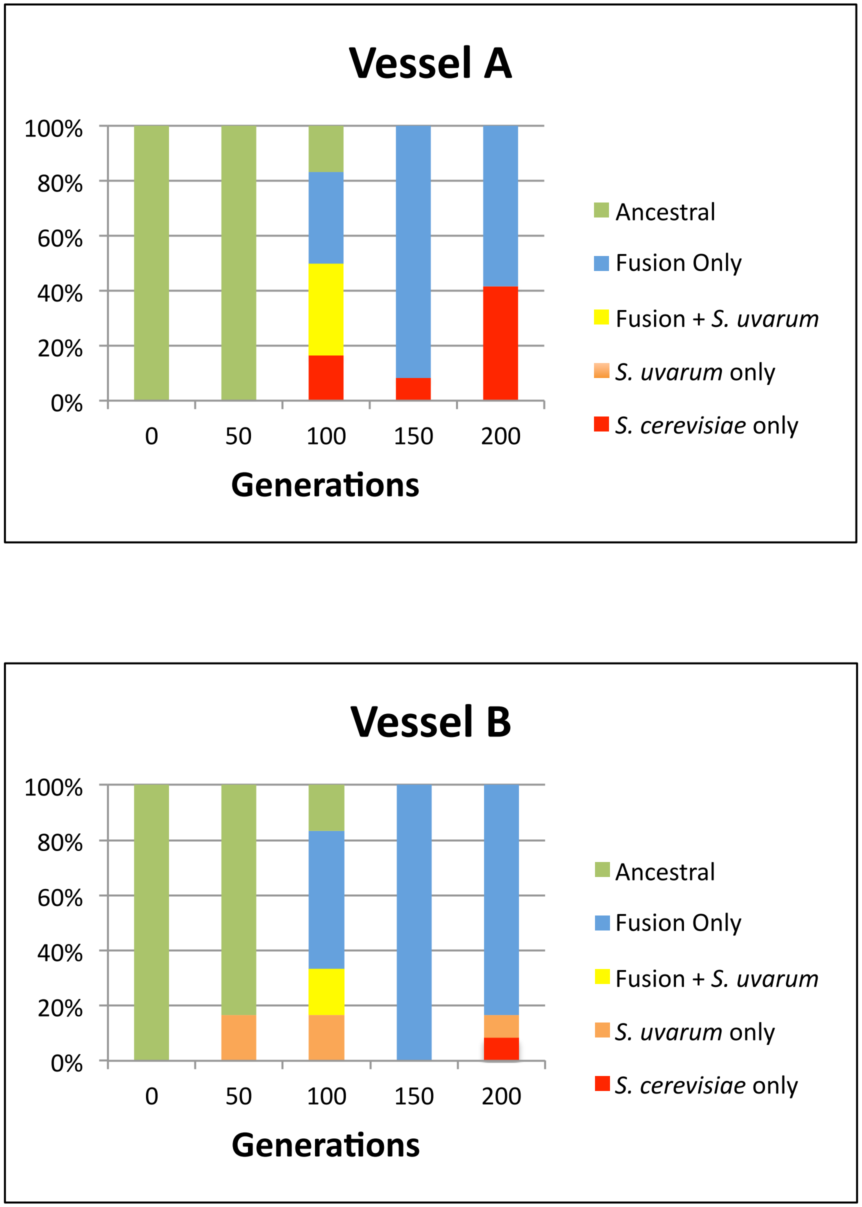

Supp. Figure 4B

```
S. uvarum       CAACATAAACAATGTCTTACAATTTTACAGGTACGCCCACGGGCGAAGGAACGGGTGGTA   60
S. cerevisiae   CAATATCAACAATGTCTTACAATTTTACAGGTACGCCTACAGGCGAAGGAACGGGTGGTA   60
Vessel A        *** ** ****************************** ** *******************   60
Vessel B        *** ** ****************************** ** *******************   60
Vessel C        *** ** ****************************** ** *******************   60

S. uvarum       ACTCATTGACCACAGACTTAAACACCCAATATGATTTGGCTAATATGGGATGGATCGGTG  120
S. cerevisiae   ACTCGTTGACAACAGATTTGAATACACAATTTGACTTGGCCAACATGGGATGGATCGGTG  120
Vessel A        **** ***** ***** ** ** ** **** *** ***** ** ****************  120
Vessel B        **** ***** ***** ** ** ** **** *** ***** ** ****************  120
Vessel C        **** ***** ***** ** ** ** **** *** ***** ** ****************  120

S. uvarum       TAGCTTCCGCAGGTGTCTGGATTATGGTGCCAGGTATCGGTCTGTTATACTCCGGTTTGT  180
S. cerevisiae   TGGCTTCAGCAGGTGTGTGGATTATGGTCCCAGGTATCGGTTTATTATATTCTGGTTTAT  180
Vessel A        * ***** ******** *********** ************ * ***** ** ***** *  180
Vessel B        * ***** ******** *********** ************ * ***** ** ***** *  180
Vessel C        * ***** ******** *********** ************ * ***** ** ***** *  180

S. uvarum       CTAGGAAAAAGCACGCTTTATCTTTGCTTTGGGCCTCGATGATGGCCTCTGCAGTGTGTA  240
S. cerevisiae   CCAGGAAAAAGCATGCTTTATCCTTGCTTTGGGCCTCGATGATGGCTTCCGCCGTGTGTA  240
Vessel A        * *********** ******** *********************** ** ** *******  240
Vessel B        * *********** ******** *********************** ** ** *******  240
Vessel C        * *********** ******** *********************** ** ** *******  240
                                            (1 100-gen)           (1 100-gen)
S. uvarum       TCTTCCAGTGGTTTTTCTGGGGGTATTCGCTGGCCTTCTCACATAACACTAGAGGTCATG  300
S. cerevisiae   TTTTCCAATGGTTTTTCTGGGGATACTCATTAGCTTTCTCACACAACACTAGAGGTAACG  300
Vessel A        * ***** ************** ** **  * ** ******** ************ * *  300
Vessel B        * ***** ************** ** **  * ** ******** ************ * *  300
Vessel C        * ***** ************** ** **  * ** ******** ************ * *  300
                        (1 100-gen)
S. uvarum       GTTTCATTGGTACTTTGGAATTCTTCGGGTTCCGTAACGTTTTGGGGGCTCCCTCCAGTG  360
S. cerevisiae   GTTTTATTGGTACCTTGGAATTCTTTGGGTTTCGTAACGTTTTAGGAGCCCCATCTAGTG  360
Vessel A        **** ******** *********** ***** *********** ** ** ** ** ****  360
Vessel B        **** ******** *********** ***** *********** ** ** ** ** ****  360
Vessel C        **** ******** *********** ***** *********** ** ** ** ** ****  360

S. uvarum       TCAGCTCATTGCCTGACATCTTATTTGCTGTTTACCAAGGTATGTTTGCCGCCGTCACCG  420
S. cerevisiae   TCAGTTCTCTTCCCGATATACTGTTTGCCGTTTACCAAGGTATGTTTGCCGCAGTCACCG  420
Vessel A        **** **  * ** ** **  * ***** *********************** *******  420
Vessel B        **** **  * ** ** **  * ***** *********************** *******  420
Vessel C        **** **  * ** ** **  * ***** *********************** *******  420
                                                   (1 100-gen)
S. uvarum       GTGCCCTAATGTTGGGTGGTGCTTGCGAAAGAGCAAGACTGTTCCCCATGATGGTGTTCT  480
S. cerevisiae   GTGCCCTAATGCTAGGTGGTGCCTGCGAGAGGGCAAGGTTGTTTCCTATGATGGTGTTCT  480
Vessel A        *********** * ******** ***** ** *****  **** ** *************  480
Vessel B        *********** * ******** ***** ** *****  **** ** *************  480
Vessel C        *********** * ******** ***** ** *****  **** ** *************  480

S. uvarum       TATTCTTATGGATGACTGTCGTCTACTGTCCAATTGCTTGCTGGGTCTGGAACGCAGAAG  540
S. cerevisiae   TGTTTTTATGGATGACTATTGTTTATTGTCCTATTGCATGCTGGGTCTGGAATGCCGAGG  540
Vessel A        * ** ************ * ** ** ***** ***** ************** ** ** *  540
Vessel B        * ** ************ * ** ** ***** ***** ************** ** ** *  540
Vessel C        * ** ************ * ** ** ***** ***** ************** ** ** *  540
                      GSY2532
                 (many, see legend)
```

Supp. Figure 4B

```
S. uvarum         GCTGGTTGGTCAAGTTAGGCAGTTTGGACTATGCTGGTGGGTTATGTGTTCATTTGACTT  600
S. cerevisiae     GTTGGTTGGTCAAATTGGGTAGCTTGGACTATGCAGGTGGTTTATGTGTCCATTTAACAT  600
Vessel A          * *********** ** ** ** *********** ***** ******** ***** ** *  600
Vessel B          * *********** ** ** ** *********** ***** ******** ***** ** *  600
Vessel C          * *********** ** ** ** *********** ***** ******** ***** ** *  600
                                           GSY2535

S. uvarum         CCGGCCATGGTGGGTTGGTCTACGCCTTGATATTGGGTAGACGTAACGACCCTGTGACAC  660
S. cerevisiae     CTGGACATGGTGGTCTAGTTTACGCTTTGATACTGGGTAAGCGTAATGACCCTGTTACAC  660
Vessel A          * ** ********  * ** ***** ****** ******  ***** ******** ****  660
Vessel B          * ** ********  * ** ***** ****** ******  ***** ******** ****  660
Vessel C          * ** ********  * ** ***** ****** ******  ***** ******** ****  660

S. uvarum         GCAAGGGAATGCCCAAGTACAAGCCACATTCCGTCACCTCCGTGGTGTTAGGAACTGTGT  720
S. cerevisiae     GTAAAGGGATGCCCAAGTACAAACCACATTCCGTCACCTCGGTGGTTTTAGGCACAGTGT  720
Vessel A          * ** ** ************** ***************** ***** ***** ** ****  720
Vessel B          * ** ** ************** ***************** ***** ***** ** ****  720
Vessel C          * ** ** ************** ***************** ***** ***** ** ****  720

S. uvarum         TTTTATGGTTCGGTTGGATGTTCTTCAACGGTGGCTCTGCCGGGAATGCCACCATACGAG  780
S. cerevisiae     TCTTATGGTTTGGTTGGATGTTCTTTAACGGAGGCTCTGCAGGTAATGCAACTATACGAG  780
Vessel A          * ******** ************** ***** ******** ** ***** ** *******  780
Vessel B          * ******** ************** ***** ******** ** ***** ** *******  780
Vessel C          * ******** ************** ***** ******** ** ***** ** *******  780

S. uvarum         CATGGTACTCTATAATGTCCACCAACTTGGCTGCTGCTTGCGGTGGGTTGACCTGGATGG  840
S. cerevisiae     CATGGTACTCTATTATGTCCACAAACTTAGCTGCTGCTTGCGGTGGCTTGACATGGATGG  840
Vessel A          ************* ******** ***** ***************** ***** *******  840
Vessel B          ************* ******** ***** ***************** ***** *******  840
Vessel C          ************* ******** ***** ***************** ***** *******  840

S. uvarum         TGATCGATTATTTCAGATCTGGTAGAAAGTGGACCACTGTCGGTCTATGTTCAGGTATCA  900
S. cerevisiae     TTATCGATTATTTCAGATGCGGAAGAAAGTGGACTACAGTTGGTTTGTGTTCAGGTATCA  900
Vessel A          * ****************  ** *********** ** ** *** * *************  900
Vessel B          * ****************  ** *********** ** ** *** * *************  900
Vessel C          * ****************  ** *********** ** ** *** * *************  900
                    (2 100-gen)                              (many, see legend)
S. uvarum         TTGCCGGTCTAGTCGGTATCACTCCAGCCGCTGGGTTTGTACCAATCTGGTCTGCCGTTG  960
S. cerevisiae     TCGCTGGCCTAGTGGGTATCACCCCAGCCGCCGGGTTCGTGCCAATCTGGTCAGCCGTTG  960
Vessel A          * ** ** ***** ******** ******** ***** ** *********** *******  960
Vessel B          * ** ** ***** ******** ******** ***** ** *********** *******  960
Vessel C          * ** ** ***** ******** ******** ***** ** *********** *******  960

S. uvarum         TCATTGGTGTGGTCACTGGTGCAGGTTGTAATCTTGCTGTTGACTTGAAGGGCTTATTAG 1020
S. cerevisiae     TCATTGGTGTGGTTACTGGTGCAGGATGTAACCTTGCTGTTGACTTAAAGAGTCTATTGC 1020
Vessel A          ************* *********** ***** ************** *** *  ****   1020
Vessel B          ************* *********** ***** ************** *** *  ****   1020
Vessel C          ************* *********** ***** ************** *** *  ****   1020

S. uvarum         GTATTGACGATGGTTTAGACTGTTACTCTATCCATGGTGTGGGTGCTTGTATTGGCTGTG 1080
S. cerevisiae     GCATCGATGATGGTCTAGATTGTTACTCTATCCATGGTGTGGGTGGTTGTATTGGTTCTG 1080
Vessel A          * ** ** ****** **** ************************* ********* * ** 1080
Vessel B          * ** ** ****** **** ************************* ********* * ** 1080
Vessel C          * ** ** ****** **** ************************* ********* * ** 1080
                                              GSY2533
                                         (many, see legend)
```

Supp. Figure 4B

```
S. uvarum        TGCTAACTGGTATCTTTGCCGCTGATTACGTCAATGCCACTGCCGGTTCCTACATCAGTC 1140
S. cerevisiae    TATTAACTGGTATCTTTGCTGCAGACTATGTAAATGCCACTGCAGGCTCTTACATTAGTC 1140
Vessel A         *  **************** ** ** ** ** *********** ** ** ***** **** 1140
Vessel B         *  **************** ** ** ** ** *********** ** ** ***** **** 1140
Vessel C         *  **************** ** ** ** ** *********** ** ** ***** **** 1140

S. uvarum        CCATCGCCGGTGGCTGGATCAACAAACACTACAAACAAGTCGGTTATCAATTGGCTGGTA 1200
S. cerevisiae    CAATTGATGGTGGCTGGATCAATCATCACTATAAACAAGTTGGTTATCAATTAGCAGGTA 1200
Vessel A         * ** *  **************  * ***** ******** *********** ** **** 1200
Vessel B         * ** *  **************  * ***** ******** *********** ** **** 1200
Vessel C         * ** *  **************  * ***** ******** *********** ** **** 1200

S. uvarum        TGTGTGCTGCGCTAGCATGGACCGTTACTGTCACATCTATATTGCTAGTGACTATGAACG 1260
S. cerevisiae    TATGCGCTGCACTAGCCTGGACTGTTACTGTCACATCTATCTTGCTTCTAACTATGAATG 1260
Vessel A         * ** ***** ***** ***** ***************** *****  * ******** * 1260
Vessel B         * ** ***** ***** ***** ***************** *****  * ******** * 1260
Vessel C         * ** ***** ***** ***** ***************** *****  * ******** * 1260

S. uvarum        CCATCCCATTTTTGAAGCTAAGATTAAGCGCCGATGAGGAAGAATTAGGTACTGATGCTG 1320
S. cerevisiae    CCATTCCATTTTTAAAACTAAGATTAAGTGCTGATGAGGAAGAATTAGGTACCGACGCTG 1320
Vessel A         **** ******** ** *********** ** ******************** ** **** 1320
Vessel B         **** ******** ** *********** ** ******************** ** **** 1320
Vessel C         **** ******** ** *********** ** ******************** ** **** 1320

S. uvarum        CTCAAATCGGTGAATTCACCTACGAGGAATCCACCGCTTATATTCCAGAACCAATCAGAT 1380
S. cerevisiae    CTCAAATTGGTGAATTTACATACGAGGAATCCACTGCTTACATCCCAGAACCAATCAGAT 1380
Vessel A         ******* ******** ** ************** ***** ** **************** 1380
Vessel B         ******* ******** ** ************** ***** ** **************** 1380
Vessel C         ******* ******** ** ************** ***** ** **************** 1380

S. uvarum        CAAGAACTTCTGCTCAAATGCCACCTCCTCATGAAACCATCGATGACAAGATCGTTGCTA 1440
S. cerevisiae    CTAAAACATCGGCACAAATGCCACCTCCTCATGAAAACATTGATGATAAGATTGTGGGTA 1440
Vessel A         * * *** ** ** ********************** *** ***** ***** ** * ** 1440
Vessel B         * * *** ** ** ********************** *** ***** ***** ** * ** 1440
Vessel C         * * *** ** ** ********************** *** ***** ***** ** * ** 1440

S. uvarum        ACACAGACGCAGAAAAGAATTCCACGCCTTCTGATGTTTCTTCTACCAAGAACACTGACC 1500
S. cerevisiae    ACACAGACGCAGAAAAGAATTCTACGCCTTCCGACGCTTCTTCTACTAAGAACACTGACC 1500
Vessel A         ********************** ******** ** * ********* ************* 1500
Vessel B         ********************** ******** ** * ********* ************* 1500
Vessel C         ********************** ******** ** * ********* ************* 1500

S. uvarum        ATATAGTATAATGATATACCCGTACAGACTATGCTCTTATTTTTTACTCATTATGTAATC 1560
S. cerevisiae    ATATAGTATAATGATATACTCAAGCAGACTATGATTTTA-TGTTTAATCTTTATGTAACG 1560
Vessel A         ******************* *   ********* * *** * **** ** ********   1560
Vessel B         ******************* *   ********* * *** * **** ** ********   1560
Vessel C         ******************* *   ********* * *** * **** ** ********   1560
```
