# Supplementary material for: Recurrent Rearrangement during Adaptive Evolution in an Interspecific Yeast Hybrid Suggests a Model for Rapid Introgression
Source: PLoS Genet. 2013 Mar 21;9(3):e1003366. doi: 10.1371/journal.pgen.1003366 (PMC3605161; doi:10.1371/journal.pgen.1003366)
Supplement: Table S1 — Primers used in this study. At the top are listed the primers used in various combinations for “genotyping”, i.e., distinguishing the S. cerevisiae MEP2 gene, S. uvarum MEP2 gene, S. cerevisiae-S. uvarum fusion MEP2 gene, and S. uvarum-S. cerevisiae “reverse fusion” MEP2 gene. Primers marked as “sequencing” were used for targeted Sanger sequencing of the PCR products that were determined to arise from a S. cerevisiae-S. uvarum fusion MEP2 gene. Primers used for Quantitative Reverse-Transcription PCR (qRT-PCR) are shown at the bottom, for the S. cerevisiae MEP2 gene, S. uvarum MEP2 gene, and for two control genes, the S. cerevisiae TFC1 and S. uvarum YDR458C genes. GSP number refers to the laboratory primer collection number. (DOCX) [file pgen.1003366.s007.docx]

**Supplementary Table 1: Primer sequences**

| **Primer name** | **GSP Number** | **Usage** | **Primer Sequence (5' to 3')** |
| --- | --- | --- | --- |
| Sc-F-MepCheck | GSP562 | Genotyping/  Sequencing | CGTTGACAACAGATTTGAATACACAAT |
| Sc-R-MepCheck | GSP563 | Genotyping | GTGTGCACAGTTAAAGTCATCGA |
| Su-F-MepCheck | GSP564 | Genotyping | CCACAGACTTAAACACCCAATATG |
| Su-R-MepCheck | GSP565 | Genotyping | GCATGCATGTGTAAAGCCGGTC |
| MEP-Seq-2R | GSP566 | Sequencing | CATTATACTATATGGTCAGTGTTC |
| qScerMEP-1 | GSP567 | qRT-PCR | GTTCTCTTCCCGATATACTGTTT |
| qScerMEP-2 | GSP568 | qRT-PCR | CAGCATGCAATAGGACAATAAAC |
| qSuvaMEP-1 | GSP569 | qRT-PCR | GCTCATTGCCTGACATCTTA |
| qSuvaMEP-2 | GSP570 | qRT-PCR | AGCAAGCAATTGGACAGTAGAC |
| qSuvaYDR458c-1 | GSP558 | qRT-PCR | TCGTTTGGTTGCCTTTGGAC |
| qSuvaYDR458c-2 | GSP559 | qRT-PCR | ACTTCTTTAGCCCCTCCTGA |
| qScerTFC1-1 | GSP571 | qRT-PCR | GATAATGTCCCCTCTGCAAGA |
| qScerTFC1-2 | GSP572 | qRT-PCR | GCGGCAGTTGGAAATCTGTA |
