# Supplementary material for: Recurrent Rearrangement during Adaptive Evolution in an Interspecific Yeast Hybrid Suggests a Model for Rapid Introgression
Source: PLoS Genet. 2013 Mar 21;9(3):e1003366. doi: 10.1371/journal.pgen.1003366 (PMC3605161; doi:10.1371/journal.pgen.1003366)
Supplement: Table S2 — Whole genome sequencing SNP analysis. Results of single nucleotide polymorphism (SNP) detection analysis from whole genome sequencing results for 200-generation evolved clones GSY2532, GSY2533, and GSY2535, performed as described in the text. Determination of conservative, semi-conservative and non-conservative amino acid changes were made according to http://www.clustal.org/download/clustalx_help.html. (PDF) [file pgen.1003366.s008.pdf]

Supplementary Table 2: SNPs identified through whole genome sequencing

Strain	Genome	Chr.	Position	Systematic and Gene Name	SNP Effect	Reference Nucleotide	SNP Nucleotide
GSY2532 (Vessel A)	*S. uvarum*	13	567,223	Sbay_13.344 (*S. uvarum* homolog of *YMR164C*/*MSS1*)	Non-synonymous, semi-conservative (G to A)	C	G
GSY2533 (Vessel B)	*S. cerevisiae*	7	28,807	*YGL251C* (*HFM1*) coding sequence	Non-synonymous, conservative (R to K)	C	T
	S. uvarum	15	354,918	Intergenic between Sbay_15.204 and Sbay_15.206 (*S. uvarum* homologs of *YHR020W* and *YHR021C*)	N/A	C	T
GSY2535 (Vessel C)	*S. cerevisiae*	4	243,445	*YDL122W* (*UBP1*) coding sequence	Synonymous (Y to Y)	C	T
	S. cerevisiae	5	405,451	*YER123W* (*YCK3*) coding sequence	Non-synonymous, conservative (L to F)	G	T
	S. cerevisiae	7	619,623	*YGR065C* (*VHT1*) coding sequence	Non-synonymous, semi-conservative (G to A)	C	G
	S. cerevisiae	14	258,697	*YNL204C* (*SPS18*) coding sequence	Non-synonymous, non-conservative (L to W)	A	C
	S. uvarum	4	608,898	Sbay_4.344 (*S. uvarum* homolog of *YBR097W*/*VPS15*)	Synonymous (S to S)	C	A
	S. uvarum	8	695,518	Sbay_8.386 (*S. uvarum* homolog of *YOR336W*/*KRE5*)	Non-synonymous, semi-conservative (D to G)	A	G
