# Supplementary material for: Recurrent Rearrangement during Adaptive Evolution in an Interspecific Yeast Hybrid Suggests a Model for Rapid Introgression
Source: PLoS Genet. 2013 Mar 21;9(3):e1003366. doi: 10.1371/journal.pgen.1003366 (PMC3605161; doi:10.1371/journal.pgen.1003366)
Supplement: Table S3 — Physiological phenotypes of the ancestral interspecific hybrid in relation to three independently evolved clones. Estimates of cell density (A600), biomass and residual glucose were derived from culturing single colony isolates of the ancestral S. cerevisiae×S. uvarum interspecific hybrid (GSY86) and 3 evolved isolates (GSY2532, GSY2533, GSY2535). Samples were removed from aerobic, ammonium-limited chemostats at steady state, D = 0.16 h−1. We used a one-way ANOVA followed by a Student-Newman-Kuels test to test for significant differences. Different superscript lettering indicates resolved significant differences between isolates; n = 3. For all strains, residual ammonium was near or below the assay detection limit (0.01 ppm). (PDF) [file pgen.1003366.s009.pdf]

Supplementary Table 3. **Physiological phenotypes of the ancestral interspecific hybrid in relation to three independently evolved clones.** Estimates of cell density, biomass and residual glucose were derived from culturing single colony isolates of the ancestral *S. cerevisiae* x *S. uvarum* interspecific hybrid (GSY86) and 3 evolved isolates (GSY2532, GSY2533, GSY2535). Samples were removed from aerobic, inorganic nitrogen-limited chemostats at steady state, D=0.16 h^{-1}. We used a one-way ANOVA followed by a Student-Newman-Kuels test to detect significant differences. Different lettering in superscript indicates resolved significant differences between isolates; n=3. For all strains, residual ammonium was near or below the assay detection limit (0.01 ppm).

**Strain**	**A600** **Mean (S.E.)**	**Dry weight** **($mg\ L^{-1}$)** **Mean (S.E.)**	**Residual Glucose** **($g\ l^{-1}$)** **Mean (S.E.)**
**GSY86**	1.04 (0.05)	737 (24.0)[a]	1.51 (0.2)
**GSY2532**	1.08 (0.02)	807 (19.5)[b]	1.69 (0.17)
**GSY2533**	1.05 (0.03)	726 (8.1)[a]	1.59 (0.26)
**GSY2535**	1.16 (0.02)	852 (12.5)[b]	1.74 (0.36)
**ANOVA**	*P*=0.8	*P*=0.002*	*P*=0.92
